# Structure and signaling mechanism of *Helicobacter pylori* transducer-like protein D

**DOI:** 10.64898/2026.01.16.699579

**Published:** 2026-01-17

**Authors:** Kailie Franco, Megan DiIorio, Adam J. Simpkin, Ronan M. Keegan, Karen Kallio, Emily Goers-Sweeney, Michelle Colbert, S. James Remington, C. Keith Cassidy, Arkadiusz W. Kulczyk, Arden Baylink

**Affiliations:** a Department of Veterinary Microbiology and Pathology, Washington State University, Pullman, WA, USA; b Institute for Quantitative Biomedicine, Rutgers University, Piscataway, NJ, USA; c Institute of Systems, Molecular and Integrative Biology, University of Liverpool, Liverpool L69 7ZB, UK; d UKRI–STFC, Rutherford Appleton Laboratory, Research Complex at Harwell, Didcot OX11 0FA, UK; e Institute of Molecular Biology, University of Oregon, Eugene, OR, US; f Department of Physics & Astronomy, University of Missouri, Columbia, MO, US; g Department of Biochemistry & Microbiology, Rutgers University, New Brunswick, NJ, US; h CryoEMcorp, Bridgewater, NJ, US; i Amethyst Antimicrobials, LLC, Pullman, WA, USA

## Abstract

Chemoreceptors, or methyl-accepting chemotaxis proteins (MCPs), are ancient and widespread prokaryotic sensors that direct taxis in response to stimuli and are attractive targets for therapeutic control of bacteria^1–4^. Decades of study have yielded substantial mechanistic insight into chemoreceptor function, but the absence of high-resolution full-length structures containing ligand-binding domains (LBD) has limited understanding of how effector sensing is structurally coupled to long-range signal transduction. Here, we present the intact structure of the chemoreceptor transducer-like protein D (TlpD) from the gastric pathogen *Helicobacter pylori*, in complex with its ligand Zn^2+^, determined by X-ray crystallography in two crystal forms at 2.4–3.0 Å. Three different conformations are captured, revealing how interactions in the ligand-binding site of the chemoreceptor zinc-binding (CZB) domain are interconnected with the distal kinase interface. Small changes at the ligand-binding site coincide with cascades of side-chain rearrangements across the dimer, distortion of the receptor coiled-coil, and conformational and dynamic shifts at the kinase interface over 140 Å away. These near-atomic resolution structures provide a framework for understanding cooperativity and allosteric communication in chemoreceptors, and establish a representative model for a widespread class of soluble chemoreceptors important in bacterial pathogenesis^2,5^.

## Introduction

Chemoreceptors are a family of protein sensors broadly conserved among prokaryotes that direct motility in response to environmental stimuli, a behavior known as chemotaxis^1–3,6^. Chemoreceptors control bacterial navigation to sense and locate nutrients, avoid harmful compounds, and identify permissive sites for colonization, thereby promoting survival^2,7–10^. A broad range of effectors have been described, including sugars, amino acids, aryl hydrocarbons, pH, metals, and reactive oxygen species (ROS), with each species possessing a repertoire of receptors suited for particular ecological niches^1,4,11^. Bacterial chemoreceptors have long served as model coiled-coil signaling assemblies and have revealed principles of sensory transduction, cooperativity, signal amplification, and adaptation that extend across diverse systems in nature^3,12–14^. Both pathogens and commensals of the gastrointestinal tract employ chemoreceptors for host colonization and persistence, suggesting these systems could be targets for antimicrobial intervention and microbiome engineering^2,8^. The sensitivity and selectivity of chemoreceptors for diverse biological stimuli, such as their ability to discriminate between chiral small molecules, has spurred the development of chemotaxis-based tools for microbe-delivered cancer therapeutics and synthetic reporter systems^15,16^.

The most prevalent and best-studied chemoreceptors are membrane-bound, elongated homodimers exemplified by the Tsr and Tar receptors possessed by *E. coli* and other *Enterobacteriaceae* (Supplemental Fig. 1)^1,4,5,17^. These receptors contain a periplasmic ligand-binding domain (LBD), transmembrane region, and an antiparallel four-helix cytosolic coiled-coil known as the methyl-accepting (MA) domain^1–3,5,6,11,17,18^. Chemoreceptors assemble into trimers of homodimers, forming extended hexagonal arrays that span hundreds of nanometers, are strongly cooperative, and enhance sensitivity by an order of magnitude (Supplemental Fig. 1)^3,19–26^. Each chemoreceptor homodimer functions as a discrete signaling unit in which ligand-sensing initiates conformational and dynamic changes that propagate over long distances, spanning 150 to 250 Å, depending on MA domain length, to the coiled-coil tips that interface with and regulate the activity of the histidine kinase CheA^1,4,27–29^. Phosphorylated CheA transfers phosphate to the response regulator CheY, which diffuses to the flagellar motor and transiently pauses or reverses rotation to induce directional changes in swimming trajectory (Supplemental Fig. 1)^3,6,28,30,31^. By modulating the frequency of swimming reorientations in response to chemical gradients, with attractants and repellents decreasing and increasing changes in swimming trajectory, respectively, bacteria achieve directed movement to favorable environments^3,6,18,32,33^.

Despite extensive study using a variety of structural, biochemical, and cellular methodologies, understanding of key mechanisms of chemoreceptor function, namely, how effector recognition is coupled to long-range allostery, cooperative homodimeric signaling, and kinase regulation, has been limited to mechanistic models without accompanying high-resolution structural data of an intact chemoreceptor and LBD. To date, only isolated LBDs or cytosolic fragments have been determined^17,26,28,34–38^. Here, we report the first structures of a cytoplasmic and fully soluble chemoreceptor, transducer-like protein D (TlpD) from *Helicobacter pylori*. These full-length chemoreceptor structures, with its native ligand bound, provide high-resolution snapshots of coordinated structural changes linking ligand-binding interactions to the conformational changes and dynamics of chemoreceptor signal transduction.

## Results & Discussion

### Structure determination of TlpD from Helicobacter pylori

To overcome the long-standing challenges associated with structural studies of transmembrane chemoreceptors, we focused on a conserved class of cytoplasmic, or “soluble” chemoreceptors that retain core signaling architecture but lack membrane-spanning segments^1,4,5^. We selected for study the protein TlpD from *H. pylori* strain J99, a CZB-regulated chemoreceptor that plays important roles in gastric colonization and is possessed by many host-associated species of bacteria^39–45^. Sequence analysis predicted TlpD to be predominantly α-helical and to contain three domains/regions: (1) an N-terminal segment of unknown structure and function, (2) a MA domain, and (3) a C-terminal CZB domain.

CZB domains are a poorly understood type of LBD that regulate chemoreceptors and diguanylate cyclases by binding a single Zn^2+^ through tetrahedral coordination via three discontiguous histidines and a cysteine thiolate^39–41,44,46–48^. We previously characterized the function of TlpD and the CZB-regulated diguanylate cyclase Z (DgcZ) from *Escherichia coli* as sensors of hypochlorous acid (HOCl), a major bactericidal ROS generated by neutrophils^40,44,48^. We showed the Cys-Zn moiety is selectively oxidized to cysteine-sulfenate through a typical S_N_2 reaction, promoting Zn^2+^ release and local unfolding of the CZB to initiate signal transduction, ultimately regulating taxis and biofilm formation, respectively^40,44,48^. More generally, as many bacteria that possess CZBs do not encounter high concentrations of HOCl, CZB domains may employ Zn^2+^ as a secondary messenger to sense effectors or conditions that perturb zinc homeostasis, such as ROS, pH, and metabolic state^39–41,43,44,46–48^.

After crystallizing the protein and collecting two sets of high-resolution diffraction data, solving these datasets was hindered by strong anisotropy, weak anomalous signal, and lack of suitable homologous models for conventional molecular replacement (see Methods, Supplemental Fig. 2). Structure determination was eventually achieved through a multi-stage molecular replacement strategy that leveraged recent advances in structure prediction combined with fragmentation of predicted models to accommodate conformational variations within the coiled-coil (Methods, Supplemental Fig. 3)^49,50^. Retrospective analysis indicates that a pronounced bend within the MA coiled-coil was the major impediment to earlier solution efforts (Supplemental Fig. 3).

For the two TlpD crystal structures, anisotropic resolution cutoffs were applied^51^: a *P*1 structure containing four chains at 2.45–3.28 Å resolution (PDB: 10EA), and a *P*2_1_2_1_2_1_ structure containing two chains at 2.92–3.07 Å (PDB: 10DZ), which capture three independent TlpD homodimers (Supplemental Fig. 3, 4, 5). The dimer structures represent possible signaling intermediates along a conformational trajectory in which progressive distortions of the Zn^2+^-binding site reflect successive steps in weakening ligand-protein interactions that culminate in Zn^2+^ release and promotion of a kinase-OFF state (Supplemental Fig. 3). In the sections that follow, we utilize the most complete and best-resolved TlpD homodimer, Chains CD from the *P*1 crystal form, as a reference to describe the overall architecture of the protein and key structural features (Supplemental Fig. 4, 5). Thereafter, we compare the three TlpD homodimers and map how changes in chemoreceptor ligand-binding coordinate with conformational and dynamic shifts in the kinase interface.

### Architecture of the TlpD homodimer

**Fig. 1.**
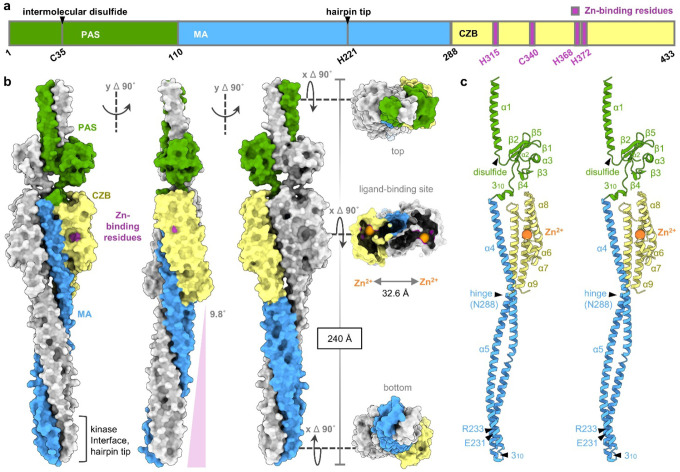
Architecture of TlpD. a. Domain organization of TlpD, with key structural features indicated. b. Crystal structure of the TlpD homodimer in six orientations (*P*1 CD), with one protomer colored as in a and the partner chain in white. The molecular surface is shown, with Zn^2+^ ions depicted as enlarged orange spheres for clarity. c. Cross-eyed stereo view of a TlpD monomer, highlighting secondary structure elements and key structural features.

**Fig. 2.**
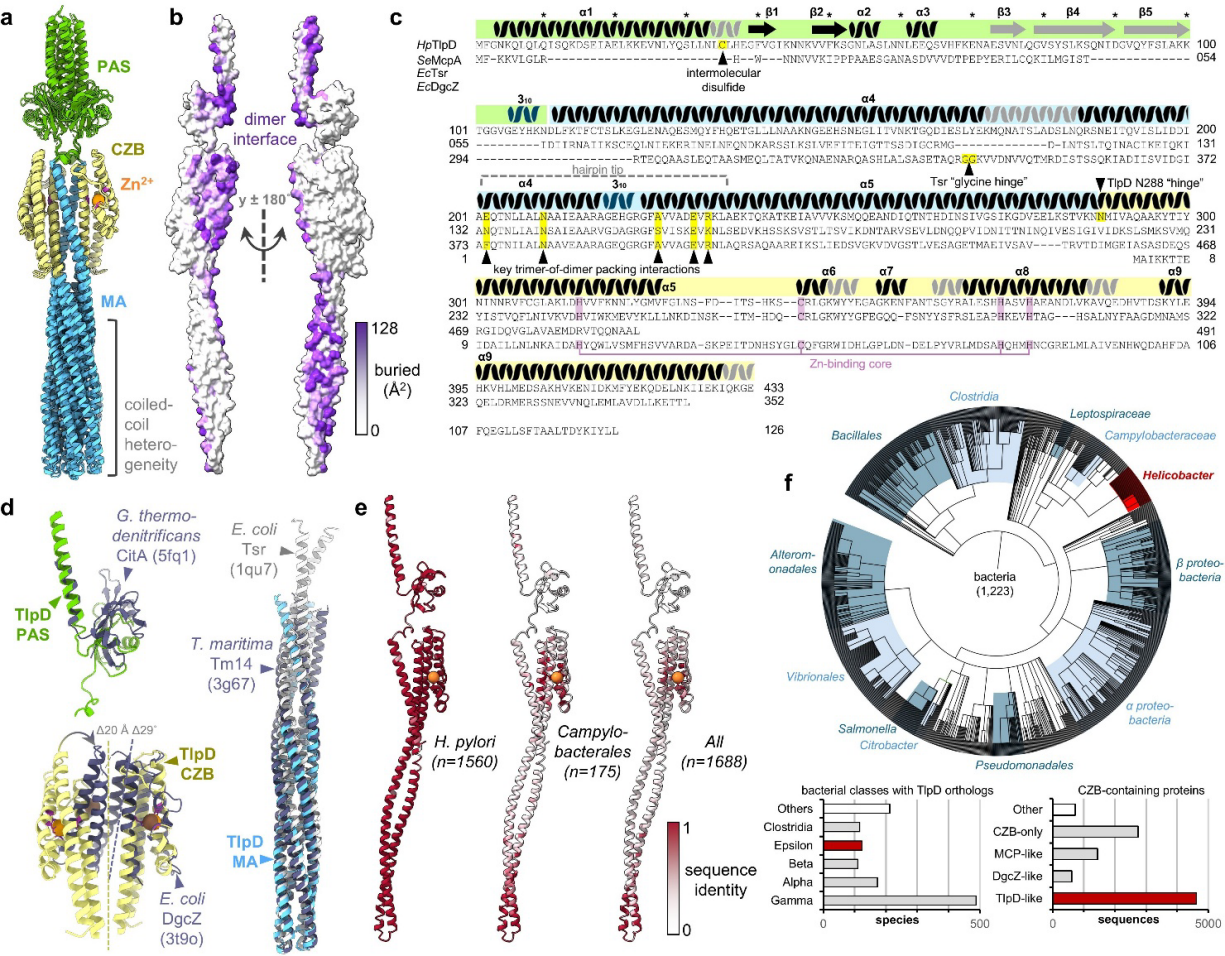
Structural comparisons and evolutionary conservation of TlpD. a. Overlay of six TlpD crystal structure chains using residues 115–135, the central core of the MA domain that threads between the CZBs. b. The TlpD homodimer interface of the *P*1 CD homodimer. c. Sequence alignment of *H. pylori* TlpD (strain J99) and related proteins *S*. Typhimurium McpA, another CZB-regulated chemoreceptor, *E. coli* Tsr, a transmembrane chemoreceptor, and E. coli DgcZ, a CZB-regulated diguanylate cyclase. Helical and strand regions in TlpD are indicated with coils and arrows, respectively, with gray indicating variable regions. Domain regions are colored as in a and Fig. 1. Pink indicates the zinc-binding residues, and key regions of structural interest are denoted with yellow highlights and black carrots. d. Overlays of relevant structures with TlpD domains, as indicated. e. Amino acid conservation across TlpD orthologs mapped onto the TlpD structure. f. Distribution of TlpD orthologs in nature. Top; phylogenetic tree of TlpD-containing organisms, bottom; major TlpD-containing clades and architecture type of CZB-containing proteins. The taxa/architecture groups to which *H. pylori* TlpD belongs are noted in red.

The TlpD homodimer is revealed to form an elongated key-like structure, 240 Å in length, in which the three domains of each protomer: an N-terminal Per-Arnt-Sim (PAS) (residues 1–110), MA (111–288), and C-terminal CZB (289–433) are intertwined along the central coiled-coil in a nearly two-fold symmetrical embrace (Fig. 1a,b, Supplemental Fig. 6). The protein contains nine α-helices, two 3_10_-helices, and five β-strands, although the secondary structure elements vary slightly across the structures, mostly in the continuity of helical regions (Fig. 1b, Supplemental Fig. 5, 6, 7)^52^. The major structural difference across the TlpD dimers is the degree of curvature of the MA domains, ranging from 1.1–11° deviation from the central axis (Fig. 1,2, Supplemental Fig. 3,5,6,8). This bending occurs at a hinge between the MA and CZB domains at residue N288 (Fig. 1b,c, Fig. 2a). The differences in the MA domains between the TlpD dimers are made obvious by superposition of each chain and its dimer partner based on the MA region located at the core of the protein (residues 115–135 of α4) with the six chains aligning within a root-mean-square deviation (RMSD) of only 2.85 Å across 375 Cα positions (Fig. 2a, Supplemental Fig. 8). We generated homodimer AlphaFold 3 (AF3) models for *H. pylori* strains J99, G27, and SS1, which possess minor differences in sequence, and found that the MA domain and coiled-coil tip in these models also exhibit the largest deviations from the experimental structures (Supplemental Fig. 9, Table S2)^53^.

By virtue of the elongated coiled-coil structure, the dimer interface is distributed along the length of the protomers and buries a substantial amount of surface area, totaling 5750 Å^2^ (Fig. 2b, Supplemental Fig. 10). Roughly 36% of residues make interdimeric contacts, with 1790 Å^2^ contributed from the N-terminal region, 3310 Å^2^ from the MA domain, and 650 Å^2^ from the CZB domain (Supplemental Fig. 10). Although the homodimer approximates two-fold symmetry, the positions and buriedness of equivalent residues vary depending on the chains analyzed (Supplemental Fig. 10).

### Domain structure of TlpD

We generated sequence alignments of TlpD with three relevant comparators: (1) the chemoreceptor McpA from *S*. Typhimurium, a CZB-regulated chemoreceptor with similar architecture to TlpD, (2) the more distantly related *E. coli* chemoreceptor Tsr, which lacks a CZB and is membrane-bound, and (3) DgcZ from *E. coli,* a CZB-regulated diguanylate cyclase (Fig. 2c). These comparisons, along with overlays of previously-determined structures, reveal both conserved and divergent features, and a potential structural basis for cellular functions of TlpD noted in prior work, which we describe below in order from the N- to C-terminus (Fig. 2c,d, Supplemental Fig. 10,11, 12).

That the N-terminal region of TlpD contains a PAS domain was unexpected and not obvious from its sequence (Fig. 1c). The TlpD PAS makes little contact with the rest of the protein, joining to the MA domain through a 3_10_ helix and close in space to the α5-α6 and α8-α9 loops of the CZB, and exhibits a large degree of flexibility and disorder evidenced by high B-factors and weak electron density (Fig. 1b,c, Supplemental Fig. 3, 4, 5). The PAS structure has no obvious ligand-binding pocket, suggesting its function could be mechanical rather than sensory (Fig. 1b, c). Neither McpA nor most TlpD orthologs outside of *Helicobacter* contain a PAS domain and its sequence and structure diverge substantially from characterized PAS families, with its closest analogue being a *Geobacter* histidine kinase (PDB 5fq1; 16% identity; DALI Z-score 6.9, Fig. 2d,e, Supplemental Fig. 11).

The native organization of TlpD in signaling arrays has not been determined, but earlier work demonstrated soluble chemoreceptors can assemble head-to-head, in which their N-terminal regions interdigitate, forming a sandwich of two arrays within the cytosol^54^. TlpD is known to localize at the cell poles, but upon exposure to oxidative stress, relocates to the cell body^5,39,41^. Interestingly, we find that the α1 helix contains an intermolecular disulfide bond at C35, which could potentially mediate redox-dependent shifts in TlpD array formation, and also explains the presence of covalently linked TlpD oligomers seen during protein purification and in earlier work (Methods, Fig. 1c, Supplemental Fig. 5)^39,40^. Another recent study showed that C-terminal truncation ablates TlpD polar localization, which, based on these structures, we expect is due to disrupting stabilizing interactions contributed by the CZB α8-α9 loop (Fig. 1c)^55^.

The central MA domain of TlpD adopts the archetypal antiparallel coiled-coil common to the chemoreceptor protein family, albeit only about half the length of that of transmembrane receptors like Tsr^29^. The N-terminal end of α4 winds down to a hairpin tip and 3_10_ helix, with its apex at H221, then folds back upon itself and ascends through α5 to join to the C-terminal CZB domain (Fig. 1a,b,c, Fig. 2d). Differences in the MA bending and supercoiling among the TlpD dimers facilitate large differences in the orientation of the coiled-coil tips relative to the positions of the other domains (Fig. 2a, Supplemental Fig. 8). In comparing the structure of the TlpD MA to previous crystal structures from the cytosolic fragment of Tsr of *E. coli*, and cytosolic receptor Tm14 of *Thermotoga maritima*, which lacks a LBD, we find all three structures adopt similar topology, especially at the kinase interface, overlaying within 1.32 Å RMSD over 85 Cα^34,56^. TlpD possesses the conserved residues E231 and R233, known to mediate the formation of trimers-of-homodimers (Fig. 1c, Fig. 2d, Supplemental Fig. 2)^23,34,35^. A key difference from Tsr and other well-studied transmembrane chemoreceptors is that TlpD does not possess the six canonical methylation sites that mediate adaptation in transmembrane chemoreceptors, consistent with the absence of CheR and CheB in *H. pylori* (Fig. 2c,d); Tm14 also lacks these sites and has been proposed to undergo methylation at other positions^5,56,57^.

TlpD is a chemoreceptor regulated by a C-terminal CZB, an architecture common to around half of all CZB-containing proteins (Fig. 2e,f, Supplementary Data 1)^44^. Until now, the only CZB structure available was that of *E. coli* DgcZ (PDB: 3t9o, 4h54), an atypical form representing about 5% of CZB proteins where the CZB is N-terminal and not coupled to an MA domain^44,46^. In DgcZ, the two CZB domains pack directly against one another to form the homodimer interface, but for chemoreceptors like TlpD, it was unknown how the CZBs could accommodate the MA coiled-coil (Fig. 2d)^40,44,46^. We find that the C-terminal CZB of TlpD adopts a compact four helix bundle formed from α5-α9, where α6-α7 are nearly continuous helices (Fig. 1c, 2c, Supplemental Fig. 5, 6, 7), and the α4 and α4′ helices of the MA domains are threaded between the two CZBs (Fig. 1b). In this configuration, the CZBs straddle the central coiled-coil and do not interface directly (Fig. 2a,d, Supplemental Fig. 12). This arrangement is achieved by tilting the two-fold symmetry axis interface by approximately 28°, pivoting the N-terminal end of α9 nearly 20 Å (Fig. 2d, Supplemental Fig. 12). Thus, the predominant homodimeric topology for CZB proteins is revealed to be fundamentally different from that of the previously characterized DgcZ^44,46^.

### Conservation and phylogenetic distribution of TlpD

Defining the phylogenetic distribution of TlpD provides insight into the evolutionary breadth and functional constraints of this chemoreceptor class. TlpD is found to be restricted to bacteria, with 1,223 species encoding identifiable homologues, distributed across diverse phyla and particularly common in *Alpha*-, *Beta*-, *Gamma*-, and *Epsilonproteobacteria*, as well as in *Clostridia*, *Bacillales*, and *Leptospiraceae* (Fig. 2f, Supplementary Data 1). Among these are many disease-causing bacteria, such as members of the genera *Helicobacter*, *Campylobacter*, *Salmonella*, *Vibrio*, and *Morganella* (Fig. 2f, Supplementary Data 1).

Mapping amino acid conservation onto the TlpD structure highlights which residues and regions are preserved across species and thus likely to be broadly important for function (Fig. 2e). All regions of TlpD are strongly conserved among *H. pylori*, but amino acid conservation decreases substantially among more distantly-related bacteria, with only the core of the CZB domain and the hairpin tip of the MA domain retained (Fig. 2e, Supplemental Fig. 9, 12). The most variable portion of the protein appears to be the N-terminal region: it is truncated or entirely absent in many species, including *M. morganii* and *S*. Typhimurium (Fig. 2c,e, Supplemental Fig. 11, Supplementary Data 1). Our AlphaFold 3 models suggest that some CZB-regulated chemoreceptors, such as those from *Campylobacter jejuni*, *Aeromonas hydrophila*, and *Vibrio cholerae*, possess an extended N-terminal helix that effectively lengthens α4 rather than the PAS domain found in *H. pylori* TlpD (Fig. 2c,e, Supplemental Fig. 11, Supplementary Data 1)^53^.

### Coordination of the Zn^2+^ ligand

**Fig. 3.**
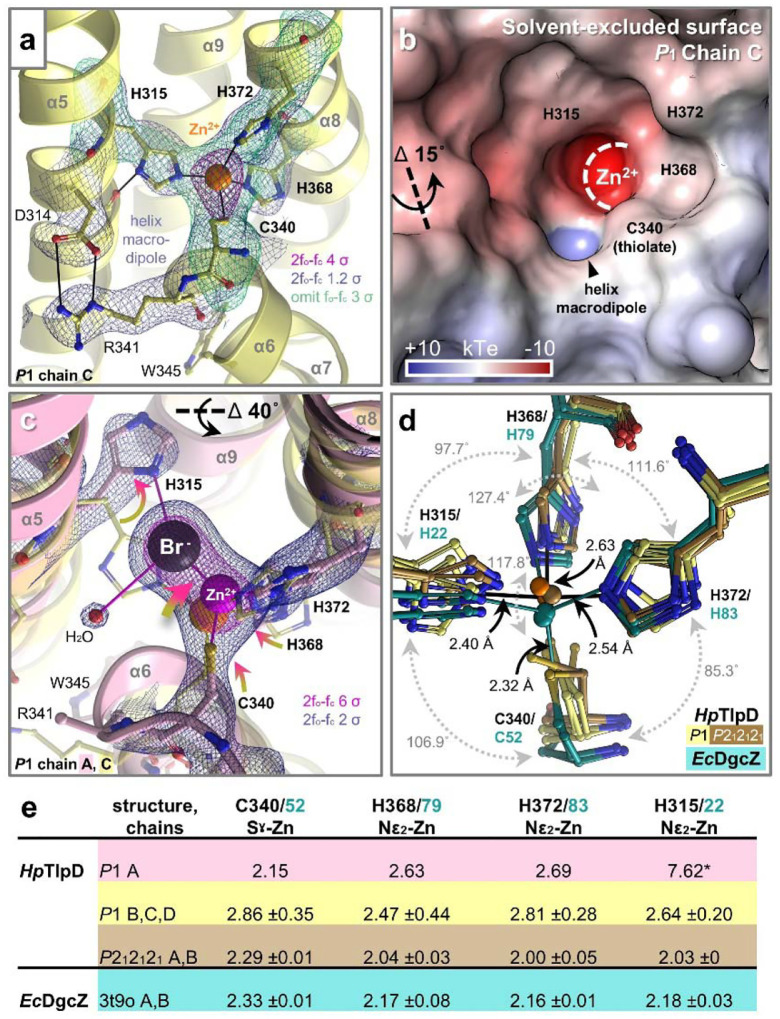
Variable coordination of the Zn^2+^ ligand within the CZB binding site. a. Ligand-binding interactions and electron density in *P*1 Chain C, as indicated. b. Electrostatic surface potential of the zinc-binding site. c. Comparison of zinc-binding sites of *P*1 Chain C (yellow) with *P*1 Chain A (opaque pink); structural shifts between Chain C and A are indicated by yellow-to-pink arrows. The bromide atom is shown as a black sphere. Electron density mesh is for Chain A. d. Overlay of zinc-binding sites from the *H. pylori* TlpD *P*1 CD homodimer chains (light yellow), *H. pylori* TlpD *P*2_1_2_1_2_1_ AB homodimer chains (dark yellow), and the two chains of the CZB domain of *E. coli* DgcZ (teal, PDB: 3t9o). TlpD residue numbers are indicated in black and DgcZ in teal. Bond lengths and angles shown in black are for *P*1 Chain C, and teal bonds for DgcZ Chain B. e. Zn^2+^ bond lengths ±STD (Å); * denotes distance, not a bond.

The mechanisms that underlie TlpD function as a Zn^2+^ sensor, as well as a sensor of HOCl via direct oxidation of the Cys-Zn redox switch, have until now lacked insights from experimental structural data^40,44,48^. Fortuitously, the 3His, 1Cys Zn^2+^-binding site of TlpD is well resolved for all chains, providing six independent views of the Zn^2+^ coordination environment (Fig. 3a, Supplemental Fig. 5, 13). Chain C of the *P*1 structure provides a particularly exquisite view of the native tetrahedral ligation of the Zn^2+^, with the metal ligand exhibiting a strong 2F_o_-F_c_ electron density peak of approximately 12 σ (Fig. 3a, Supplemental Fig. 13). The surrounding helices of the CZB domain cradle the Zn^2+^ ion at the center of the bundle, coordinated by H315 (α5), C340 (α6), and H368 and H372 (α8) (Fig. 1b, 2d); although H367 and H337 are also nearby, neither participates in ligation. A salt bridge between D314 and R341 couples the helices α5 and α6 together, and W345 acts as a hydrophobic anchor, securing the α6-α7 segment to the CZB core (Fig. 3a).

The CZB domain forms a mostly buried ligand-binding pocket accessible to solvent through a narrow channel (Fig. 3a,b). A notable feature of the ligand-binding environment is a strong helix macrodipole of +10^−5^ kT·e, generated by the backbone NHs of R341 and L342 at the start of α6, forming a small cavity with strong positive electrostatic potential adjacent to the C340 Sγ (Fig. 3b). This macrodipole might serve two functions. First, it could increase the affinity of the protein for Zn^2+^ by raising the energetic penalty for metal release. Second, it may help bind, orient, and activate an HOCl hydroxyl for nucleophilic attack by C340, analogous to the ROS-binding strategies used by thiol peroxidases^48,58^. At the back of the pocket, proximal to the Zn^2+^, a halide-binding site is observed, with density in the *P*1 dimers consistent with Cl^−^ or Br^−^, with 2F_o_-F_c_ peaks of approximately 14–18 σ, the latter being introduced through heavy atom soaks of the crystal for the purpose of anomalous phasing (Supplemental Fig. 13). Prior quantum mechanical calculations suggested HOCl binds with the Cl^−^ positioned externally to the pocket, but the presence of halogens within the binding site indicates the opposite orientation is possible, such that the Cl leaving group engages the Zn^2+^ while the OH group aligns with the helix macrodipole (Supplemental Fig. 13)^40,44,48^.

The signaling state of TlpD is thought to be controlled by Zn^2+^ occupancy, where the ligand-bound form promotes CheA activation (kinase-ON) and the *apo* receptor inhibits CheA autophosphorylation (kinase-OFF)^40,44^. Interestingly, transitional intermediates in this process may be modeled by halogen invasion of the binding site, providing insight into how disruption of zinc coordination impacts the structure of the CZB domain. At some binding sites, halides are present and participate in unusual zinc ligation geometry, forming a pseudo-trigonal bipyramidal configuration, even though the halide is around 4 Å from the Zn^2+^, which is too distant for stable coordination (Supplemental Fig. 13)^59^. The most intrusive of these interactions occurs in Chain A of the *P*1 structure, where a Br^−^ displaces H315, and the lack of the Nε_2_-Zn^2+^ bond is accompanied by a series of structural rearrangements: the H315 side-chain swings toward α9, the Zn^2+^ shifts by 1.5 Å, and C340, H372, and H386 follow and maintain zinc coordination, while α6 and α8 piston toward the N-terminal domain, altering their register relative to α5 and α9, and these changes in α6 render the R341 side-chain disordered and sever its salt bridge with D314 (Fig. 3c, Supplemental Fig. 13).

Whereas the typical zinc ligation bond lengths for His Nε_2_ and Cys Sγ atoms are 2.0 and 2.3 Å, respectively, the TlpD zinc sites exhibit a large degree of variation (Fig. 3c,d,e, Supplemental Fig. 13)^59^. Progressively from most distorted to most ideal ligation geometry are: (1) the *P*1 Chain A, (2) the *P*1 Chains B, C, D, and (3) the *P*2_1_2_1_2_1_ Chains A and B, which also exhibit the coordination geometry most similar to that of the DgcZ CZB (Fig. 3d). Together, these structures show that the CZB zinc site is metastable, which may impart structural plasticity necessary for the sensor to reversibly engage the Zn^2+^ ligand for signaling functions. Additionally, the differences at the zinc-binding site enable a high-resolution view into how changes in the LBD are propagated to the MA domain and kinase interface, which we discuss below.

### Structural communication between the binding site and kinase interface

**Fig. 4.**
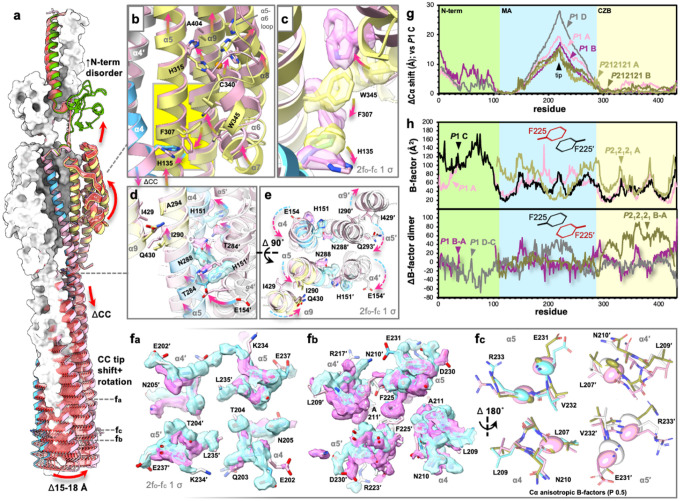
Structural connections between the Zn^2+^-binding site and kinase interface. a. An overlay of the zinc-strained homodimer (*P*1 CD, colored by domain as in Fig. 1a and white, respectively) and the zinc-disrupted (*P*1 AB, pink) is shown, with a morph between the two structures highlighting the conformational changes in red. Key structural changes are indicated with red arrows; CC denotes coiled-coil. b-e. Close-up views of structural changes in respective regions of TlpD, as indicated. Arrows highlight shifts from the zinc-strained conformation to the zinc-disrupted conformation; yellow-to-pink arrows in the CZB region and blue-to-pink arrows in the MA and MA-interacting regions. f_a-c_. To compare the CC tip region, overlays were performed using only the local kinase interface region residues 204–234. Shown are changes in local structure, electron density, and b-factor at the hairpin tip; electron density is shown as light blue and pink surface for the zinc-strained and zinc-disrupted structures, respectively. g. Changes in Cα position compared to reference chain *P*1 C; residues 115–135 were used for the structural overlays. h. Crystallographic B-factor for chains with the one trans/+80° Phe225 rotamer (top) and their partner chains with a Phe225′ rotamer of g /−30° (bottom).

Earlier studies of chemoreceptor signaling have found biochemical and cellular evidence of piston-like shifts, axial rotations, scissor movements, order-to-disorder transitions, and other structural changes as the basis by which ligand-binding regulates signal transduction at the distant coiled-coil tips^18,23,29,36,37,60–67^. Here, we visualize high-resolution snapshots of TlpD in different conformations and elucidate the structural linkages between distortions at the ligand-binding site, the MA domain, and the hairpin tip (Fig. 4a, Supplemental Fig. 14, 15). The *P*2_1_2_1_2_1_ AB and *P*1 CD dimers, hereafter referred to as the “zinc-intact” and “zinc-strained” conformations, respectively, are nominally similar in positioning of the CZB and in connection to the MA domain (Supplemental Fig. 13, 14). In contrast, the *P*1 AB dimer, which has lost coordination by H315, and which we now refer to as the “zinc-disrupted” conformation, shows a cascade of rearrangements that provide structural insights into the long-range control of the kinase interface (Fig. 4a, Supplemental Fig. 13, 14, 15). Superimposing the zinc-strained and zinc-disrupted conformations reveals extensive global structural changes; the loss of the full tetrahedral zinc coordination in the CZB is accompanied by modifications to the N-terminal PAS interface, which may promote its destabilization, and also alterations in MA coiled-coil supercoiling and bending that shift the hairpin tips by 18–20 Å (Fig. 4a, Supplemental Fig. 14, 15).

To understand the transition from the zinc-strained to the zinc-disrupted conformation, we mapped a series of side-chain reorientations that emanate from the disrupted zinc ligation site within the CZB (Fig. 3c, Fig. 4b, Supplemental Fig. 15). Ablation of the H315-Zn^2+^ bond is accompanied by a 1–2 Å movement in α5 and α9, and a substantial rearrangement involving approximately 45 residues that shift by 4–5 Å. This repositioning wedges W345 (α6) deeper into the helix bundle, imposing a steric clash with F307 (α5), which flips to occlude H135 (α4), and the side-chain reorients, thereby transmitting the structural perturbation from the LBD to α4 of the MA coiled-coil (Fig. 4b,c).

Alongside the conformational changes at the N-terminal end of α4, incremental deviations in helical positioning accrue in the C-terminal direction over a 15-residue span, with these distortions becoming pronounced at H151 (α4) which rotates away from N288 (α5′), thereby disrupting their intermolecular hydrogen bond and facilitating bending of the MA domain (Fig. 4d,e). Viewed down the central coiled-coil axis toward the tips, supercoiling is reduced through counterclockwise rotation of α4 and α5 in one protomer and clockwise rotation in its partner, with bending modulated by steric interference between E154 (α4′) and T284 (α5) (Fig. 4e, Supplemental Fig. 14). These MA domain rotations are mechanically coupled back to the α9 helices of both CZB domains via steric collisions between I290 (α5) and I429 (α9), with opposite helical twists in the disrupted versus strained conformations (Fig. 4e). This enables conformational asymmetry to occur where, in the zinc-disrupted CZB site (*P*1 Chain A), the dimer partner (*P*1 Chain B) displays ligation geometry similar to the zinc-strained conformation (Fig. 3d,e, Fig. 4e). Hence, these coordinated rotational rearrangements between six helices define an allosteric network between the LBD, MA domain, and across the receptor dimer that might serve to mediate negative cooperativity between the two ligand-binding sites, a well-documented property of chemoreceptor homodimers^38,60^.

The alterations in the MA domain proximal to the CZB domain coincide with large structural changes in the coiled-coil tips, with residues E204–K234 rotating together as a module while retaining their local structure (Fig. 4, Supplemental Fig. 14, 15, 16). Several residues near the key positions R233 and E231 tolerate locally unfavorable interactions that may help maintain a low energetic barrier between signaling conformations, such as the bundle containing T204 (α4) and L235 (α5), which provides no hydrogen bonding groups to satisfy the T204 side-chain hydroxyl (Fig. 4f_a_). The rotameric Phe switch described for transmembrane receptors is also present in TlpD, with the 2-fold symmetry of the dimer interface poising F225 and F225’ to compete for the same interior packing site, causing them to adopt different rotamers: one trans/+80° and the other g /−30°, shifted away and toward the hairpin tip, respectively (Fig. 4f_b_)^65^. Each dimer partner is asymmetric in both conformation and disorder, as evidenced by plotting Cα shifts and B-factors, and chains with similar positioning of F225 share similar patterns of order and disorder (Fig. 4g,h). The anisotropic B-factors in the trimerization segment containing D230 and R233 reveal differences in both the magnitude and orientation of atomic displacement ellipsoids, indicating distinct directions and amplitudes of thermal motion across the different TlpD conformations (Fig. 4f_c_). The zinc-intact conformation exhibits small thermal ellipsoids, whereas the zinc-strained conformation shows larger ellipsoids that angle in a similar direction, indicating translation back and forth along the plane of the α5 and α5’ helices. Interestingly, the zinc-disrupted conformation also shows larger ellipsoids than the zinc-intact, but the ellipsoids are angled to be oriented along the α4 and α5 plane (Fig. 4f_c_). Among the three TlpD conformations, the kinase interface displays progressive disorder as a function of weakening of the ligand-binding interactions, with the zinc-intact conformation showing the greatest stability and the zinc-disrupted conformation the least order (Fig. 4h, Supplemental Fig. 4).

## Conclusion

### A model for long range allostery in chemoreceptors mediated through ligand binding

Bacterial chemoreceptors are a well-studied exemplar of long coiled-coil signaling systems, within a broader class that includes the sarcomeric scaffold titin, golgins, and pericentriolar scaffold proteins at the Golgi and centrosomes, and nuclear lamins, all of which use extended α-helical rods to couple local binding or mechanical events to distal functional outputs^68,69^. Within this well-characterized signaling architecture, however, a persistent gap has been a lack of high-resolution structural information linking detection of stimuli to signaling control. The structures presented here provide high-resolution views of a soluble chemoreceptor in multiple conformational states and define a structural linkage between the distal ligand-binding site and the kinase-interacting tips. While these snapshots do not establish the temporal sequence of signaling events, they inform a model for how ligand binding biases long-range conformational and dynamic properties to regulate signal output. These structural insights establish a mechanistic foundation for long-range allostery in CZB-regulated chemoreceptors and offer a conceptual framework for understanding how extended coiled-coil domains convert local ligand-binding events into distal functional responses.

## Methods

### Cloning and purification of recombinant TlpD from H. pylori strain J99

Plasmids of the pBH vector series encoding *H. pylori* TlpD (strain J99; NCBI protein ID WP_000467796), containing an ampicillin resistance marker and a T7-driven expression cassette, were synthesized by GenScript. Recombinant plasmids were introduced into ArcticExpress DE3* *E. coli* cells (Agilent) via heat-shock transformation and plated on LB agar supplemented with ampicillin. Following incubation overnight at 37 °C, individual colonies were selected for protein expression.

Single colonies were used to inoculate 4 × 2 mL LB cultures containing ampicillin and grown overnight at 37 °C with shaking. These starter cultures were then used to inoculate 4 ×1 L LB+ampicillin cultures, which were grown at 37 °C until reaching an OD of 0.6–0.8. Cultures were subsequently shifted to 10 °C, induced with 1 mM IPTG, and allowed to express protein overnight at 10 °C. After approximately 16 h, cells were collected by centrifugation at 5,000 × g and 4 °C.

Cell pellets were resuspended in chilled lysis buffer consisting of 50 mM HEPES (pH 7.9), 300 mM NaCl, 10% glycerol, 10 mM imidazole, and 0.5 mM TCEP. Cells were maintained on ice and lysed by sonication, followed by centrifugation at 23,000 × g to remove insoluble material. The clarified lysate was loaded onto a gravity-flow Ni-NTA agarose column (Qiagen) pre-equilibrated with lysis buffer and incubated for 30–60 min, with the lysate passed over the resin twice to enhance binding. The column was washed extensively with lysis buffer until no detectable protein remained in the flow-through, assessed Bradford assay. Bound protein was eluted using lysis buffer supplemented with 300 mM imidazole.

For removal of the N-terminal His tag, eluted fractions were dialyzed against lysis buffer and incubated overnight with TEV protease (1 mg per 20 mL eluate; New England Biolabs). The cleaved protein was subsequently repurified. Final purity was assessed by SDS-PAGE, and appropriate fractions were pooled and further purified by size-exclusion chromatography using an ÄKTA FPLC system. Purified proteins were flash-frozen in liquid nitrogen and stored at −80 °C.

### Protein crystallography

Early crystallization trials with full-length TlpD from *H. pylori* strain J99 produced thin, needle-like crystals under numerous conditions (Supplemental Fig. 2a), and occasionally small wedge-shaped crystals (Supplemental Fig. 2b). Systematic optimization of conditions led to well-diffracting crystals growing in a condition of 0.1 M sodium malonate, 0.5 mM TCEP, and 12% PEG 3350, at 25 °C with protein at 8 mg/ml (Supplemental Fig. 2c). The TlpD crystals were soaked with glycerol as a cryoprotectant, and some were also soaked with bromine for potential SAD or MAD phasing before flash freezing for cryogenic X-ray diffraction analysis.

High-resolution diffraction data were obtained from two TlpD crystals, yielding datasets at 2.4–3.0 Å resolution and putative assignments to space groups *P*1 and *P*2_1_2_1_2_1_ (Supplemental Fig. 2d,e)^70,71^. Both datasets displayed substantial anisotropy. Attempts at SAD or MAD phasing failed as datasets collected at the Zn^2+^ or Br^−^ absorption edges produced insufficient anomalous signal. Molecular replacement proved successful using a homodimeric model of TlpD generated using ABCFold and Boltz-1, which was segmented with Slice N’ Dice to enable independent MR searches for different portions of the molecule^49,50,72^. This approach yielded promising domain placements in the *P*1 dataset (Supplemental Fig. 3)^73,74^. Extensive manual rebuilding ultimately produced a complete solution containing two TlpD homodimers (Supplemental Fig. 3a). This in turn guided solution of the *P*2 2 2 dataset, which contains a single homodimer in the asymmetric unit (Supplemental Fig. 3b).

To address the impact of anisotropy, which contributed to noisy electron density and elevated B-factors, we applied the STARANISO pipeline and determined appropriate resolution cutoffs for the *P*1 and *P*2 2 2 datasets to be 2.45–3.28 Å, and 2.92–3.07, respectively, based on CC_1/2_ > 0.3^51^. Although the final R- and B-factors remain higher than typically expected at these nominal resolutions, the resulting maps are substantially improved, and most regions of the protein are well resolved in all six chains (Supplemental Table 1). Clear electron density is present for the CZB domains, the Zn^2+^ ligands and coordinating residues, and the kinase-interacting regions (Supplemental Figs. 3–5). The primary exception is the N-terminal region, which varies among the three homodimers: it is modeled in three of the four chains in the *P*1 structure, while in the *P*2 2 2 structure crystal packing prevents it from folding, leaving this region unresolved (Supplemental Fig. 5)^75,76^.

### TlpD Conservation and Phylogenetics

Filtration of the protein database generated by Perkins *et al*. in 2021 was performed to identify soluble non-redundant CZB-containing proteins across diverse genera and within the order *Campylobacterales*^42^. The sequence of *H. pylori* TlpD J99 was also used as a search query in the software Geneious Prime 2025 with default cutoffs, searching through all notated *H. pylori* sequences as of August 2025. Sequences were aligned using MUSCLE and Phylogenetic trees were constructed with phyloT.

## Supplementary Material

1

## References

[1] Ortega Á., Zhulin I. B. & Krell T. Sensory Repertoire of Bacterial Chemoreceptors. Microbiology and Molecular Biology Reviews 81, e00033–17 (2017). 10.1128/MMBR.00033-17 PMC5706747 29070658

[2] Zhou B., Szymanski C. M. & Baylink A. Bacterial chemotaxis in human diseases. Trends in Microbiology https://doi.org/10.1016/j.tim.2022.10.007 (2022) doi:10.1016/j.tim.2022.10.007. PMC11238666 36411201

[3] Hazelbauer G. L., Falke J. J. & Parkinson J. S. Bacterial chemoreceptors: high-performance signaling in networked arrays. Trends in Biochemical Sciences 33, 9–19 (2008). 18165013 10.1016/j.tibs.2007.09.014 PMC2890293

[4] Gumerov V. M., Ulrich L. E. & Zhulin I. B. MiST 4.0: a new release of the microbial signal transduction database, now with a metagenomic component. Nucleic Acids Res 52, D647–D653 (2023). 10.1093/nar/gkad847 PMC10767990 37791884

[5] Collins K. D., Lacal J. & Ottemann K. M. Internal Sense of Direction: Sensing and Signaling from Cytoplasmic Chemoreceptors. Microbiology and Molecular Biology Reviews 78, 672–684 (2014). 25428939 10.1128/MMBR.00033-14 PMC4248653

[6] Matilla M. A. & Krell T. Chemoreceptor-based signal sensing. Current Opinion in Biotechnology 45, 8–14 (2017). 28088095 10.1016/j.copbio.2016.11.021

[7] Keegstra J. M., Carrara F. & Stocker R. The ecological roles of bacterial chemotaxis. Nat Rev Microbiol 1–14 (2022) doi:10.1038/s41579-022-00709-w. 35292761

[8] Matilla M. A. & Krell T. The effect of bacterial chemotaxis on host infection and pathogenicity. FEMS Microbiol Rev 42, (2018). 10.1093/femsre/fux052 29069367

[9] Schavemaker P. E. & Lynch M. Flagellar energy costs across the tree of life. eLife 11, e77266 (2022). 35881430 10.7554/eLife.77266 PMC9323006

[10] Matilla M. A., Gavira J. A. & Krell T. Accessing nutrients as the primary benefit arising from chemotaxis. Current Opinion in Microbiology 75, 102358 (2023). 37459734 10.1016/j.mib.2023.102358

[11] Bi S. & Lai L. Bacterial chemoreceptors and chemoeffectors. Cell. Mol. Life Sci. 72, 691–708 (2015). 25374297 10.1007/s00018-014-1770-5 PMC11113376

[12] Berg H. C. & Brown D. A. Chemotaxis in Escherichia coli analysed by Three-dimensional Tracking. Nature 239, 500–504 (1972). 4563019 10.1038/239500a0

[13] Hazelbauer G. L. Bacterial Chemotaxis: The Early Years of Molecular Studies. Annu Rev Microbiol 66, 285–303 (2012). 22994495 10.1146/annurev-micro-092611-150120 PMC3989901

[14] Parkinson J. S. Classic Spotlight: Dawn of the Molecular Era of Bacterial Chemotaxis. Journal of Bacteriology 198, 1796–1796 (2016). 27298281 10.1128/JB.00297-16 PMC4907109

[15] Bi S., Pollard A. M., Yang Y., Jin F. & Sourjik V. Engineering Hybrid Chemotaxis Receptors in Bacteria. ACS Synth. Biol. 5, 989–1001 (2016). 27285081 10.1021/acssynbio.6b00053

[16] Gholami A., Mohkam M., Soleimanian S., Sadraeian M. & Lauto A. Bacterial nanotechnology as a paradigm in targeted cancer therapeutic delivery and immunotherapy. Microsyst Nanoeng 10, 113 (2024). 39166136 10.1038/s41378-024-00743-z PMC11333603

[17] Glenn S. J. Bacterial vampirism mediated through taxis to serum. eLife 12, RP93178 (2024). 38820052 10.7554/eLife.93178 PMC11142651

[18] Reyes G. I., Flack C. E. & Parkinson J. S. The structural logic of dynamic signaling in the Escherichia coli serine chemoreceptor. Protein Sci 33, e5209 (2024). 39555666 10.1002/pro.5209 PMC11571029

[19] Muok A. R. Atypical chemoreceptor arrays accommodate high membrane curvature. Nat Commun 11, 5763 (2020). 33188180 10.1038/s41467-020-19628-6 PMC7666581

[20] Burt A. Complete structure of the chemosensory array core signalling unit in an E. coli minicell strain. Nat Commun 11, 743 (2020). 32029744 10.1038/s41467-020-14350-9 PMC7005262

[21] Qin Z. & Zhang P. Studying bacterial chemosensory array with CryoEM. Biochemical Society Transactions 49, 2081–2089 (2021). 34495335 10.1042/BST20210080 PMC8589424

[22] Yang W. & Briegel A. Use of Cryo-EM to Study the Structure of Chemoreceptor Arrays In Vivo. in Bacterial Chemosensing: Methods and Protocols (ed. Manson M. D.) 173–185 (Springer, New York, NY, 2018). doi:10.1007/978-1-4939-7577-8_16. 29429092

[23] Yang W. In Situ Conformational Changes of the Escherichia coli Serine Chemoreceptor in Different Signaling States. mBio 10, e00973–19 (2019). 31266867 10.1128/mBio.00973-19 PMC6606802

[24] Yang W., Alvarado A., Glatter T., Ringgaard S. & Briegel A. Baseplate variability of Vibrio cholerae chemoreceptor arrays. Proceedings of the National Academy of Sciences 115, 13365–13370 (2018). 10.1073/pnas.1811931115 PMC6310817 30541885

[25] Briegel A. New Insights into Bacterial Chemoreceptor Array Structure and Assembly from Electron Cryotomography. Biochemistry 53, 1575–1585 (2014). 24580139 10.1021/bi5000614 PMC3985956

[26] Cassidy C. K. Structure of the native chemotaxis core signaling unit from phage E-protein lysed E. coli cells. mBio 14, e00793–23. 37772839 10.1128/mbio.00793-23 PMC10653900

[27] Li M. & Hazelbauer G. L. Core unit of chemotaxis signaling complexes. Proceedings of the National Academy of Sciences 108, 9390–9395 (2011). 10.1073/pnas.1104824108 PMC3111312 21606342

[28] Bi S. & Sourjik V. Stimulus sensing and signal processing in bacterial chemotaxis. Current Opinion in Microbiology 45, 22–29 (2018). 29459288 10.1016/j.mib.2018.02.002

[29] Alexander R. P. & Zhulin I. B. Evolutionary genomics reveals conserved structural determinants of signaling and adaptation in microbial chemoreceptors. Proceedings of the National Academy of Sciences 104, 2885–2890 (2007). 10.1073/pnas.0609359104 PMC1797150 17299051

[30] Parkinson J. S. Signaling mechanisms of HAMP domains in chemoreceptors and sensor kinases. Annu Rev Microbiol 64, 101–122 (2010). 20690824 10.1146/annurev.micro.112408.134215

[31] Yi T.-M., Huang Y., Simon M. I. & Doyle J. Robust perfect adaptation in bacterial chemotaxis through integral feedback control. Proceedings of the National Academy of Sciences 97, 4649–4653 (2000). 10.1073/pnas.97.9.4649 PMC18287 10781070

[32] Frangipane G. Invariance properties of bacterial random walks in complex structures. Nat Commun 10, 2442 (2019). 31164651 10.1038/s41467-019-10455-y PMC6547659

[33] Grognot M. & Taute K. M. A multiscale 3D chemotaxis assay reveals bacterial navigation mechanisms. Commun Biol 4, 669 (2021). 34083715 10.1038/s42003-021-02190-2 PMC8175578

[34] Boyd A., Kendall K. & Simon M. I. Structure of the serine chemoreceptor in Escherichia coli. Nature 301, 623–626 (1983). 6402709 10.1038/301623a0

[35] Cassidy C. K. CryoEM and computer simulations reveal a novel kinase conformational switch in bacterial chemotaxis signaling. eLife 4, e08419 (2015). 26583751 10.7554/eLife.08419 PMC6746300

[36] Bartelli N. L. & Hazelbauer G. L. Differential backbone dynamics of companion helices in the extended helical coiled-coil domain of a bacterial chemoreceptor. Protein Sci 24, 1764–1776 (2015). 26257396 10.1002/pro.2767 PMC4622210

[37] Bi S., Jin F. & Sourjik V. Inverted signaling by bacterial chemotaxis receptors. Nat Commun 9, 2927 (2018). 30050034 10.1038/s41467-018-05335-w PMC6062612

[38] Scott W. G. Refined Structures of the Ligand-binding Domain of the Aspartate Receptor from *Salmonella typhimurium*. Journal of Molecular Biology 232, 555–573 (1993). 8345523 10.1006/jmbi.1993.1411

[39] Collins K. D. The Helicobacter pylori CZB Cytoplasmic Chemoreceptor TlpD Forms an Autonomous Polar Chemotaxis Signaling Complex That Mediates a Tactic Response to Oxidative Stress. Journal of Bacteriology 198, 1563–1575 (2016). 27002127 10.1128/JB.00071-16 PMC4959281

[40] Perkins A., Tudorica D. A., Amieva M. R., Remington S. J. & Guillemin K. Helicobacter pylori senses bleach (HOCl) as a chemoattractant using a cytosolic chemoreceptor. PLoS Biol 17, e3000395 (2019). 31465435 10.1371/journal.pbio.3000395 PMC6715182

[41] Behrens W. Localisation and protein-protein interactions of the Helicobacter pylori taxis sensor TlpD and their connection to metabolic functions. Sci Rep 6, 23582 (2016). 27045738 10.1038/srep23582 PMC4820699

[42] Rolig A. S., Shanks J., Carter J. E. & Ottemann K. M. Helicobacter pylori Requires TlpD-Driven Chemotaxis To Proliferate in the Antrum. Infect Immun 80, 3713–3720 (2012). 22802346 10.1128/IAI.00407-12 PMC3457577

[43] Huang J. Y., Sweeney E. G., Guillemin K. & Amieva M. R. Multiple Acid Sensors Control Helicobacter pylori Colonization of the Stomach. PLOS Pathogens 13, e1006118 (2017). 28103315 10.1371/journal.ppat.1006118 PMC5245789

[44] Perkins A. A Bacterial Inflammation Sensor Regulates c-di-GMP Signaling, Adhesion, and Biofilm Formation. mBio 12, e00173–21 (2021). 34154415 10.1128/mBio.00173-21 PMC8262984

[45] Cooper K. G. HilD-regulated chemotaxis proteins contribute to Salmonella Typhimurium colonization in the gut. mBio 16, e00390–25. 39998229 10.1128/mbio.00390-25 PMC11980550

[46] Zähringer F., Lacanna E., Jenal U., Schirmer T. & Boehm A. Structure and Signaling Mechanism of a Zinc-Sensory Diguanylate Cyclase. Structure 21, 1149–1157 (2013). 23769666 10.1016/j.str.2013.04.026

[47] Draper J., Karplus K. & Ottemann K. M. Identification of a chemoreceptor zinc-binding domain common to cytoplasmic bacterial chemoreceptors. J Bacteriol 193, 4338–4345 (2011). 21725005 10.1128/JB.05140-11 PMC3165512

[48] Zumwalt L., Perkins A. & Ogba O. M. Mechanism and Chemoselectivity for HOCl-Mediated Oxidation of Zinc-Bound Thiolates. ChemPhysChem 21, 2384–2387 (2020). 32915482 10.1002/cphc.202000634

[49] Elliott L. G., Simpkin A. J. & Rigden D. J. ABCFold: easier running and comparison of AlphaFold 3, Boltz-1, and Chai-1. Bioinformatics Advances 5, vbaf153 (2025). 40708869 10.1093/bioadv/vbaf153 PMC12287924

[50] Simpkin A. J. Slice’N’Dice: maximizing the value of predicted models for structural biologists. Acta Crystallogr D Struct Biol 81, 105–121 (2025). 39976565 10.1107/S2059798325001251 PMC11883665

[51] STARANISO anisotropy & Bayesian estimation server. https://staraniso.globalphasing.org/cgi-bin/staraniso.cgi.

[52] Hekkelman M. L., Salmoral D. Á., Perrakis A. & Joosten R. P. DSSP 4: FAIR annotation of protein secondary structure. Protein Science 34, e70208 (2025). 40671631 10.1002/pro.70208 PMC12268231

[53] Abramson J. Accurate structure prediction of biomolecular interactions with AlphaFold 3. Nature 630, 493–500 (2024). 38718835 10.1038/s41586-024-07487-w PMC11168924

[54] Briegel A. Structure of bacterial cytoplasmic chemoreceptor arrays and implications for chemotactic signaling. eLife 3, e02151 (2014). 24668172 10.7554/eLife.02151 PMC3964821

[55] Lopez-Magaña R. & Ottemann K. M. The Helicobacter pylori TlpD cytoplasmic chemoreceptor requires an intact C-terminus for polar localization and function. 2024.11.08.622596 Preprint at 10.1101/2024.11.08.622596 (2025). PMC13001251 41660828

[56] Pollard A. M., Bilwes A. M. & Crane B. R. The structure of a soluble chemoreceptor suggests a mechanism for propagating conformational signals. Biochemistry 48, 1936–1944 (2009). 19149470 10.1021/bi801727m PMC2695681

[57] Liu X. & Ottemann K. M. Methylation-Independent Chemotaxis Systems Are the Norm for Gastric-Colonizing Helicobacter Species. J Bacteriol 204, e0023122 (2022). 35972258 10.1128/jb.00231-22 PMC9487461

[58] Perkins A., Nelson K. J., Parsonage D., Poole L. B. & Karplus P. A. Peroxiredoxins: guardians against oxidative stress and modulators of peroxide signaling. Trends Biochem Sci 40, 435–445 (2015). 26067716 10.1016/j.tibs.2015.05.001 PMC4509974

[59] Rulísek L. & Vondrásek J. Coordination geometries of selected transition metal ions (Co2+, Ni2+, Cu2+, Zn2+, Cd2+, and Hg2+) in metalloproteins. J Inorg Biochem 71, 115–127 (1998). 9833317 10.1016/s0162-0134(98)10042-9

[60] Parkinson J. S., Hazelbauer G. L. & Falke J. J. Signaling and sensory adaptation in Escherichia coli chemoreceptors: 2015 update. Trends Microbiol 23, 257–266 (2015). 25834953 10.1016/j.tim.2015.03.003 PMC4417406

[61] Hoffman M. C., Li M., Hazelbauer G. L. & Schlau-Cohen G. S. A chemoreceptor conformational equilibrium controlled by signaling inputs. Proceedings of the National Academy of Sciences 122, e2505872122 (2025). 10.1073/pnas.2505872122 PMC12280922 40632558

[62] Ottemann K. M., Xiao W., Shin Y.-K. & Koshland D. E. A Piston Model for Transmembrane Signaling of the Aspartate Receptor. Science 285, 1751–1754 (1999). 10481014 10.1126/science.285.5434.1751

[63] Ferris H. U., Zeth K., Hulko M., Dunin-Horkawicz S. & Lupas A. N. Axial helix rotation as a mechanism for signal regulation inferred from the crystallographic analysis of the *E. coli* serine chemoreceptor. Journal of Structural Biology 186, 349–356 (2014). 24680785 10.1016/j.jsb.2014.03.015

[64] Akkaladevi N., Bunyak F., Stalla D., White T. A. & Hazelbauer G. L. Flexible Hinges in Bacterial Chemoreceptors. Journal of Bacteriology 200, 10.1128/jb.00593-17 (2018). PMC5809691 29229700

[65] Ortega D. R. A phenylalanine rotameric switch for signal-state control in bacterial chemoreceptors. Nat Commun 4, 2881 (2013). 24335957 10.1038/ncomms3881 PMC4310728

[66] Flack C. E. & Parkinson J. S. Structural signatures of Escherichia coli chemoreceptor signaling states revealed by cellular crosslinking. Proceedings of the National Academy of Sciences 119, e2204161119 (2022). 10.1073/pnas.2204161119 PMC9282233 35787052

[67] Flack C. E. & Parkinson J. S. Bacterial Chemoreceptors Transmit Stimulus Signals Through Coupled Entropic Switches. Journal of Molecular Biology 438, 169614 (2026). 10.1016/j.jmb.2025.169614 PMC13046427 41448350

[68] Woolfson D. N. Understanding a protein fold: The physics, chemistry, and biology of α-helical coiled coils. J Biol Chem 299, 104579 (2023). 36871758 10.1016/j.jbc.2023.104579 PMC10124910

[69] Lupas A. N. & Bassler J. Coiled Coils - A Model System for the 21st Century. Trends Biochem Sci 42, 130–140 (2017). 27884598 10.1016/j.tibs.2016.10.007

[70] Processing serial synchrotron crystallography diffraction data with DIALS. in Methods in Enzymology vol. 709 207–244 (Academic Press, 2024). 39608945 10.1016/bs.mie.2024.10.004

[71] Agirre J. The CCP4 suite: integrative software for macromolecular crystallography. Acta Cryst D 79, 449–461 (2023). 10.1107/S2059798323003595 PMC10233625 37259835

[72] Wohlwend J. Boltz-1 Democratizing Biomolecular Interaction Modeling. bioRxiv 2024.11.19.624167 (2025) doi:10.1101/2024.11.19.624167.

[73] Emsley P., Lohkamp B., Scott W. G. & Cowtan K. Features and development of Coot. Acta Cryst D 66, 486–501 (2010). 20383002 10.1107/S0907444910007493 PMC2852313

[74] Adams P. D. PHENIX: a comprehensive Python-based system for macromolecular structure solution. Acta Crystallogr D Biol Crystallogr 66, 213–221 (2010). 20124702 10.1107/S0907444909052925 PMC2815670

[75] Meng E. C. UCSF ChimeraX: Tools for structure building and analysis. Protein Science 32, e4792 (2023). 37774136 10.1002/pro.4792 PMC10588335

[76] Schrödinger LLC. The PyMOL Molecular Graphics System, Version 3.0.

[77] Williams C. J. MolProbity: More and better reference data for improved all-atom structure validation. Protein Sci 27, 293–315 (2018). 29067766 10.1002/pro.3330 PMC5734394

