# Supplementary material for: Structure and signaling mechanism of Helicobacter pylori transducer-like protein D
Source: bioRxiv. 2026 Jan 17:2026.01.16.699579. Preprint. [Version 1] doi: 10.64898/2026.01.16.699579 (PMC12871147; doi:10.64898/2026.01.16.699579)
Supplement: 1 [file NIHPP2026.01.16.699579V1-supplement-1.pdf]

bioRxiv preprint doi: https://doi.org/10.64898/2026.01.16.699579; this version posted January 17, 2026. The copyright holder for this preprint (which was not certified by peer review) is the author/funder, who has granted bioRxiv a license to display the preprint in perpetuity. It is made available under aCC-BY 4.0 International license.

691 **Supplemental Figures**

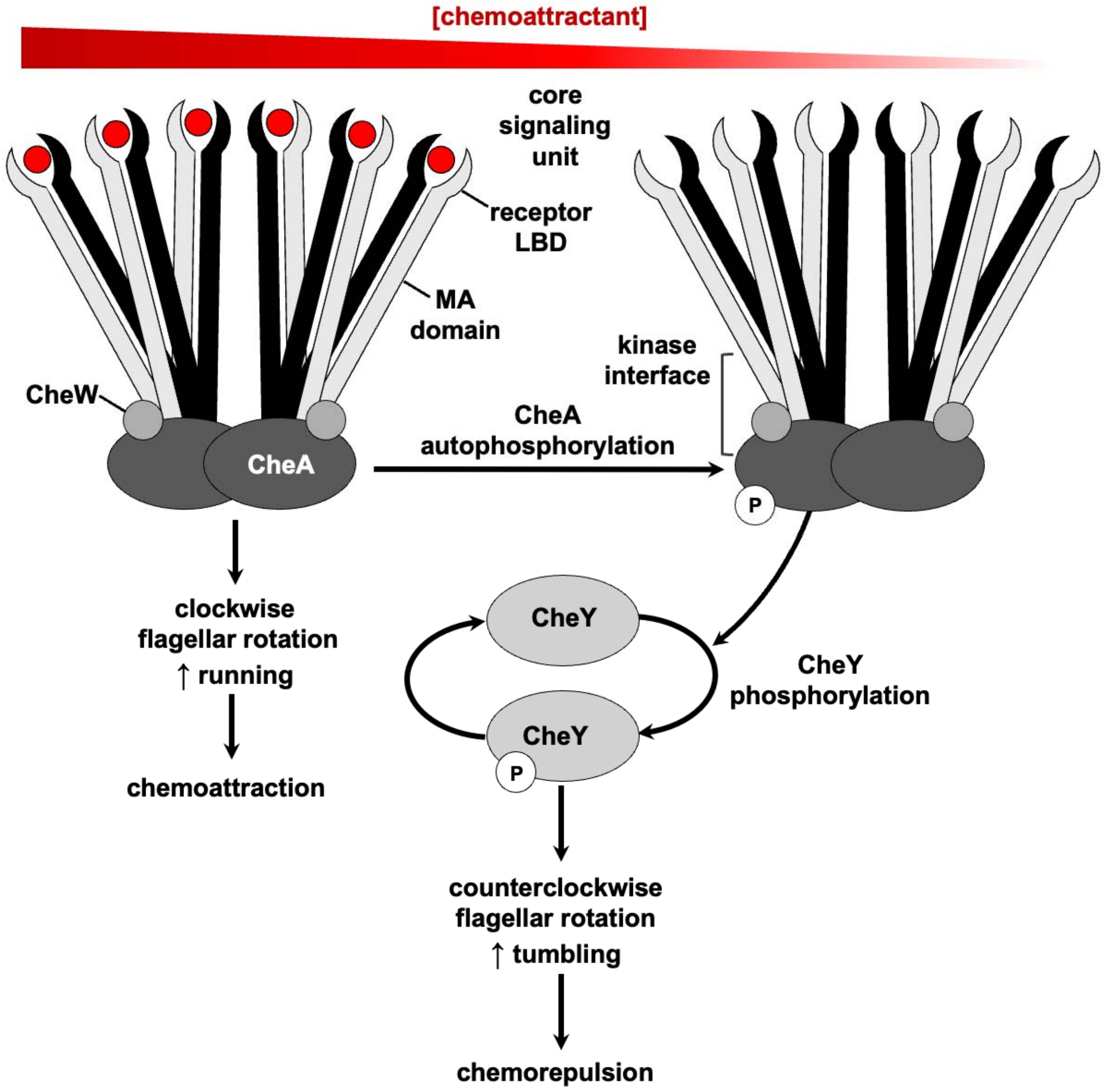

692

bioRxiv preprint doi: https://doi.org/10.64898/2026.01.16.699579; this version posted January 17, 2026. The copyright holder for this preprint (which was not certified by peer review) is the author/funder, who has granted bioRxiv a license to display the preprint in perpetuity. It is made available under aCC-BY 4.0 International license.

Supplemental Fig. 1 Overview of chemotactic signaling. Much of our current understanding of chemoreceptor signaling derives from studies in *Escherichia coli*, although diverse chemotaxis systems exist in nature that deviate from this canonical paradigm. In the *E. coli* system, chemoreceptors (black and gray rods) detect gradients of chemoeffectors (attractant shown in red) and assemble with the adaptor protein CheW and the cytosolic histidine kinase CheA to form the core signaling unit. Binding of a chemoattractant to the ligand-binding domain suppresses CheA autophosphorylation, resulting in reduced levels of phosphorylated CheY (CheY-P), sustained counterclockwise flagellar rotation, and running behavior, promotion chemoattraction. In contrast, the *apo* receptor state or binding of a chemorepellent enhances CheA autophosphorylation, leading to increased CheY-P production. Diffusible CheY-P interacts with the flagellar motor to promote clockwise rotation, inducing tumbling and swimming reorientation, which biases movement away from unfavorable stimuli/conditions, known as chemorepulsion.

bioRxiv preprint doi: https://doi.org/10.64898/2026.01.16.699579; this version posted January 17, 2026. The copyright holder for this preprint (which was not certified by peer review) is the author/funder, who has granted bioRxiv a license to display the preprint in perpetuity. It is made available under aCC-BY 4.0 International license.

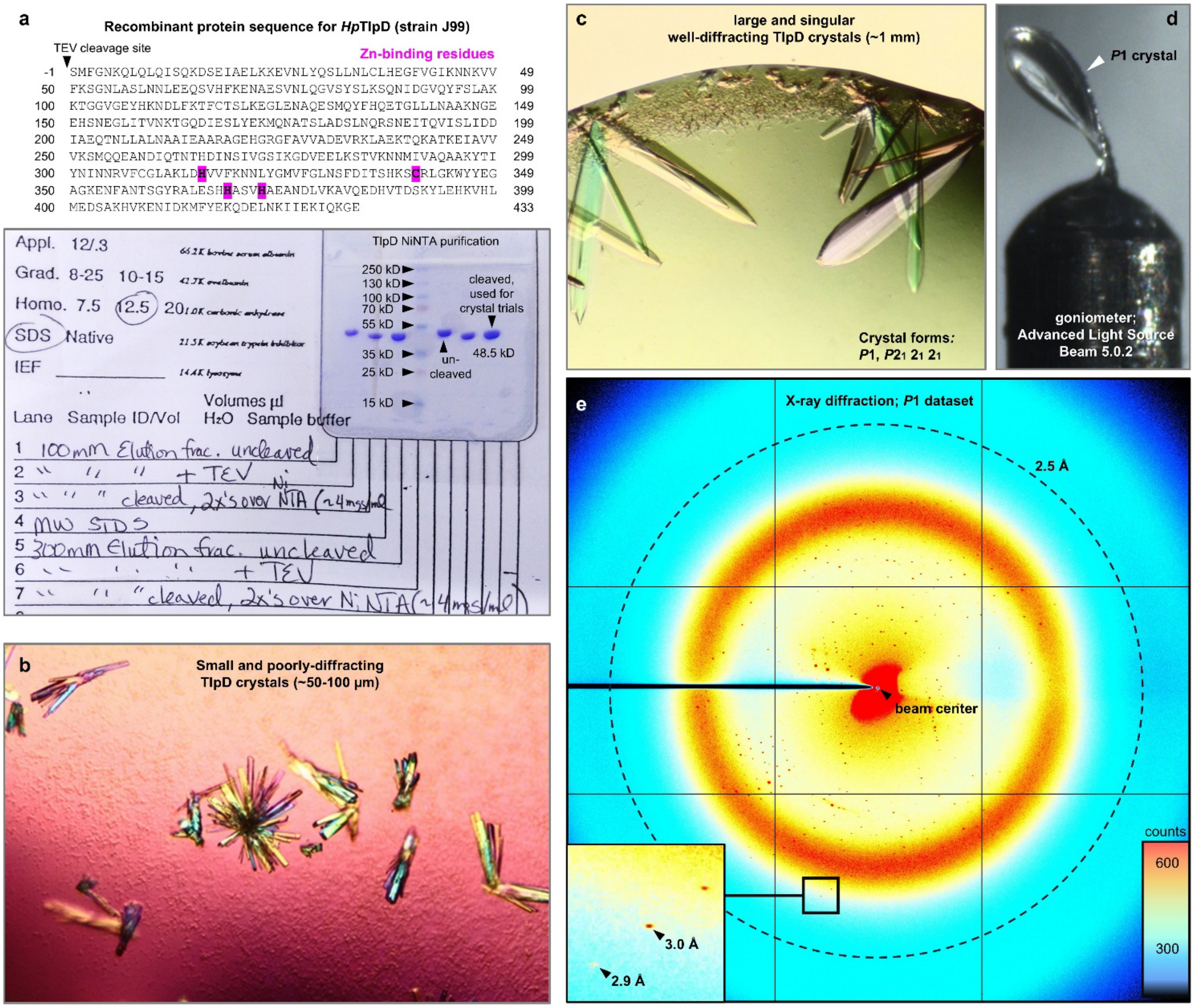

Supplemental Fig. 2. Crystallization of *Helicobacter pylori* TlpD strain J99. a. Full amino acid sequence of the crystallized protein. *H. pylori* TlpD from strain J99 was expressed with an N-terminal 6x His-tag containing a TEV cleavage site; an N-terminal Ser remains following TEV cleavage. Zinc-binding residues are highlighted in pink. Shown bottom are purification steps from Ni-NTA purification and TEV-cleavage for the protein used for crystallography. b. Shown is a representative image of the typical small clusters of poorly-diffracting crystals that TlpD readily forms in a variety of PEG and ammonium sulfate-based crystallization conditions. c. An image of the drop in which large and well-diffracting TlpD crystals grew that yielded the datasets in this study. d. An image of the *P*1 crystal (harvested from c) that yielded the initial

bioRxiv preprint doi: https://doi.org/10.64898/2026.01.16.699579; this version posted January 17, 2026. The copyright holder for this preprint (which was not certified by peer review) is the author/funder, who has granted bioRxiv a license to display the preprint in perpetuity. It is made available under aCC-BY 4.0 International license.

solution. The crystal was flash frozen and x-ray diffraction data collected under cryo conditions.

e. A representative diffraction image from the crystal in d.

bioRxiv preprint doi: https://doi.org/10.64898/2026.01.16.699579; this version posted January 17, 2026. The copyright holder for this preprint (which was not certified by peer review) is the author/funder, who has granted bioRxiv a license to display the preprint in perpetuity. It is made available under aCC-BY 4.0 International license.

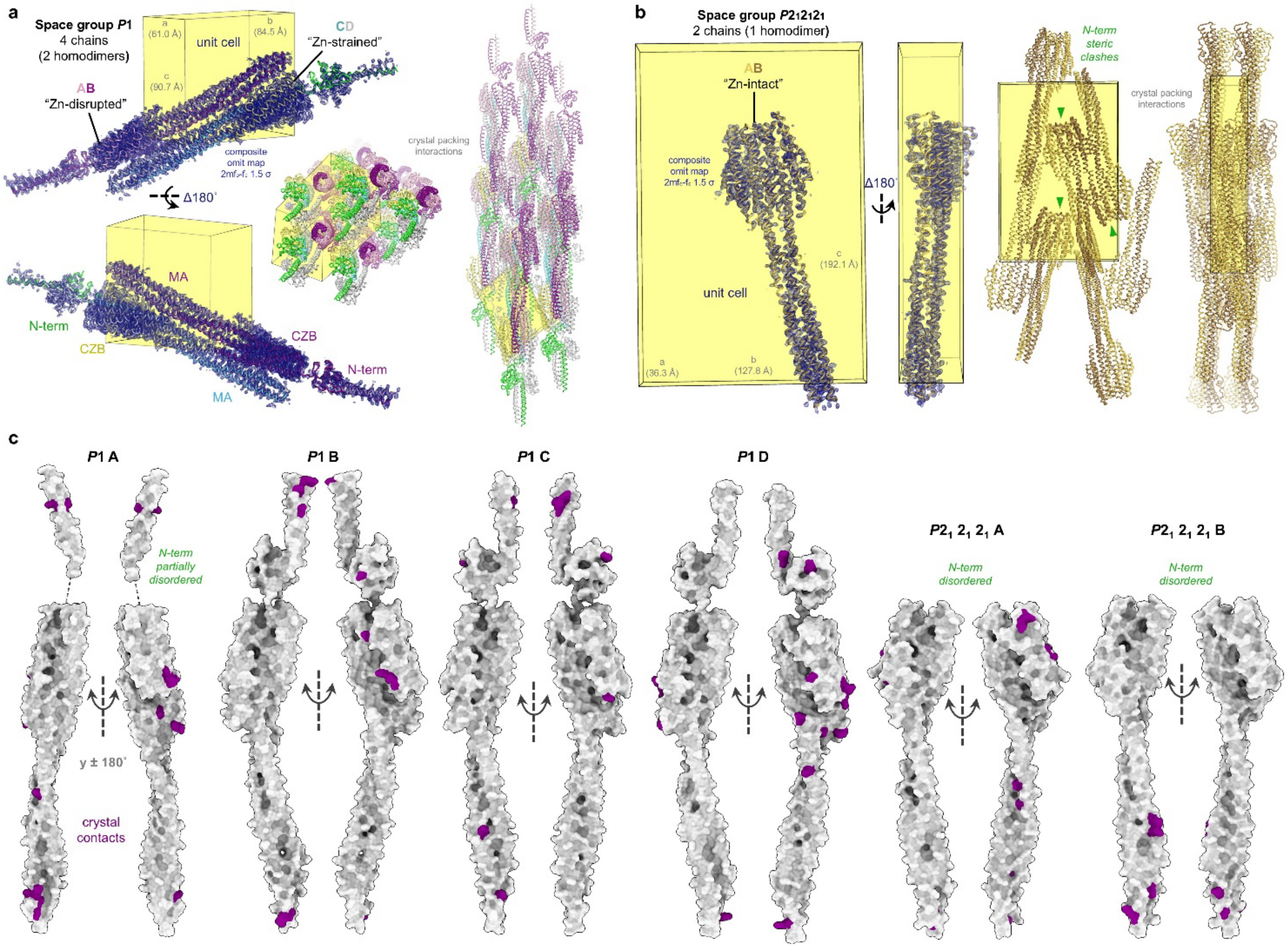

Supplemental Fig. 3. Solution of *Helicobacter pylori* TlpD in two crystal forms. a. Shown are the crystal arrangements for the *P*1 crystal structure of TlpD, consisting of two homodimers within the asymmetric unit that we refer to as the conformations "zinc-disrupted" (Chains AB, light and dark pink, respectively) and "zinc-strained" (Chains CD, colored by domain, as indicated, and gray, respectively). The N-terminal PAS is green, the MA domain is light blue, and the CZB domain is yellow. Electron density (dark blue mesh, $2F_o$-F_c) is from a composite omit map generated with 5% of the atoms removed. For all chains, the N-terminal PAS domain is poorly ordered and exhibits weak electron density. b. The crystal arrangements for the $P2_12_12_1$ TlpD crystal structure composed of Chains AB (gold and brown). Arrows (green) note crystal contact interactions that pose steric clashes for a folded N-terminal region, and hence this region

bioRxiv preprint doi: https://doi.org/10.64898/2026.01.16.699579; this version posted January 17, 2026. The copyright holder for this preprint (which was not certified by peer review) is the author/funder, who has granted bioRxiv a license to display the preprint in perpetuity. It is made available under aCC-BY 4.0 International license.

of the structure is disordered and unresolved in this crystal form. c. Residues involved in crystal contacts for each chain are colored in magenta.

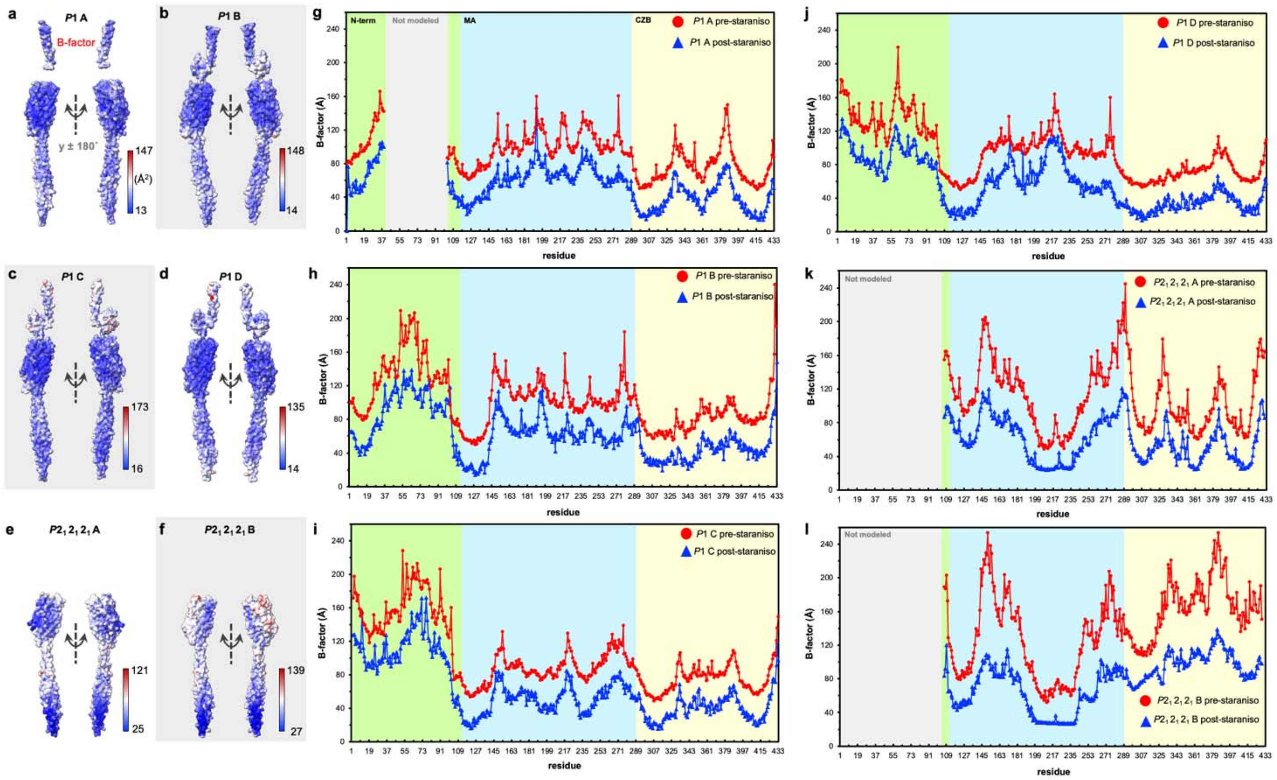

Supplemental Fig. 4. Analysis of crystallographic B-factors. a-f. Each TlpD chain is shown with molecular surface representation colored by B-factor as indicated. g-l. Plots showing B-factor per residue before (red circles) and after (blue triangles) Staraniso data processing. Domain designations are colored as in Fig. 1a., or gray for regions not modeled.

bioRxiv preprint doi: https://doi.org/10.64898/2026.01.16.699579; this version posted January 17, 2026. The copyright holder for this preprint (which was not certified by peer review) is the author/funder, who has granted bioRxiv a license to display the preprint in perpetuity. It is made available under aCC-BY 4.0 International license.

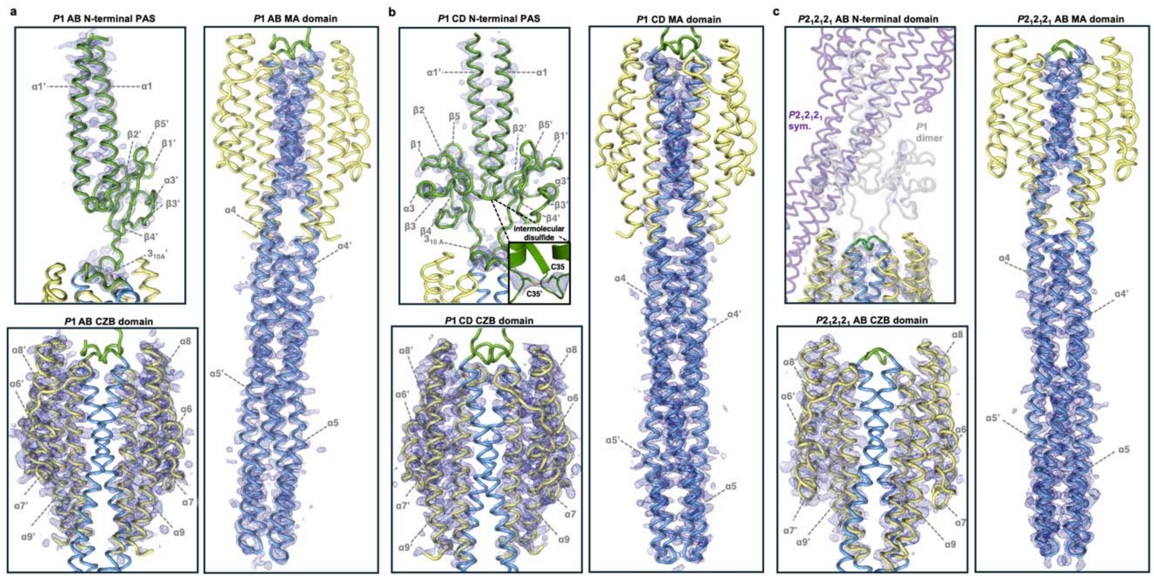

Supplemental Fig. 5. Electron density quality of the TlpD homodimers. a-c. Electron density for each TlpD homodimer is shown for different regions of the protein, as indicated. Dark blue mesh represents $2F_o$-F_c omit map density at 1 σ calculated with 5% of atoms missing. with the protein colored by domain: green for N-terminal PAS, light blue for the MA domain, and yellow for the CZB domain. For the $P2_1 2_1 2_1$ N-terminal PAS, a symmetry mate is shown in purple, and the N-terminal region of the P1 CD homodimer is modeled in gray, to illustrate the steric clash that prevents a folded N-terminus in this structure.

bioRxiv preprint doi: https://doi.org/10.64898/2026.01.16.699579; this version posted January 17, 2026. The copyright holder for this preprint (which was not certified by peer review) is the author/funder, who has granted bioRxiv a license to display the preprint in perpetuity. It is made available under aCC-BY 4.0 International license.

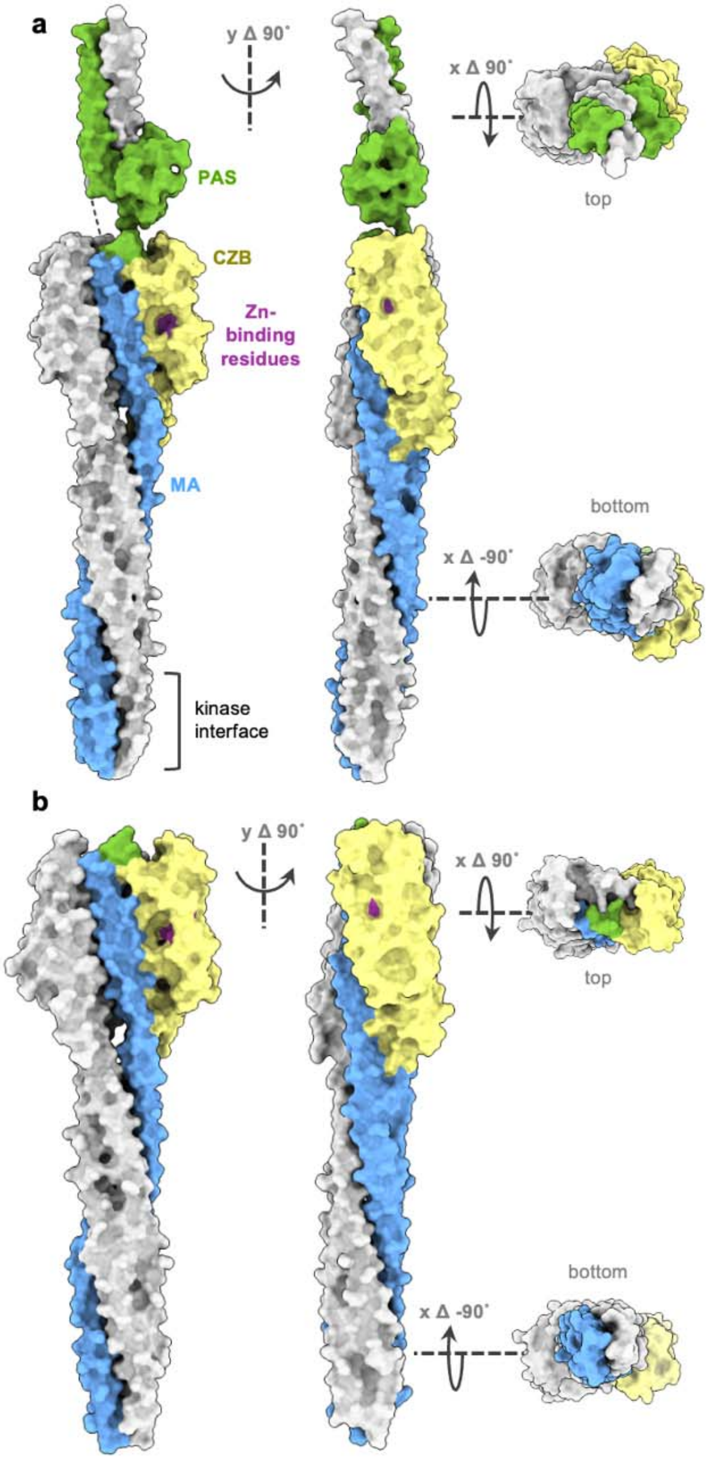

Supplemental Fig. 6. Overall structures of the *P*1 AB and *P*$2_1 2_1 2_1$ AB TlpD homodimers. a-b. Crystal structure shown as molecular surface of the *P*1 TlpD AB homodimer and *P*$2_1 2_1 2_1$ AB homodimer, respectively, colored as in 1a.

bioRxiv preprint doi: https://doi.org/10.64898/2026.01.16.699579; this version posted January 17, 2026. The copyright holder for this preprint (which was not certified by peer review) is the author/funder, who has granted bioRxiv a license to display the preprint in perpetuity. It is made available under aCC-BY 4.0 International license.

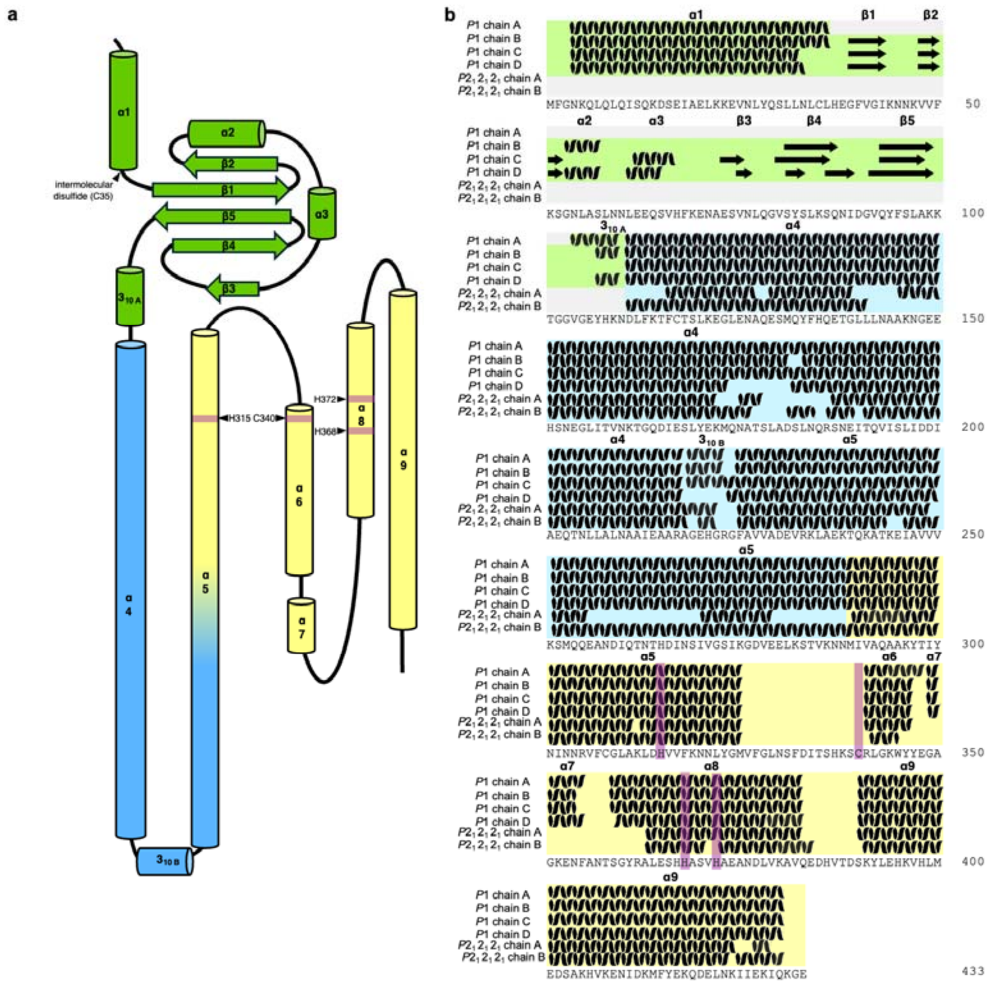

Supplemental Fig. 7. Secondary structure and consensus topology of TlpD a. A consensus topology map and key structural features of TlpD. b. Secondary structure for each chain by residue, colored by domain as in 1a.

bioRxiv preprint doi: https://doi.org/10.64898/2026.01.16.699579; this version posted January 17, 2026. The copyright holder for this preprint (which was not certified by peer review) is the author/funder, who has granted bioRxiv a license to display the preprint in perpetuity. It is made available under aCC-BY 4.0 International license.

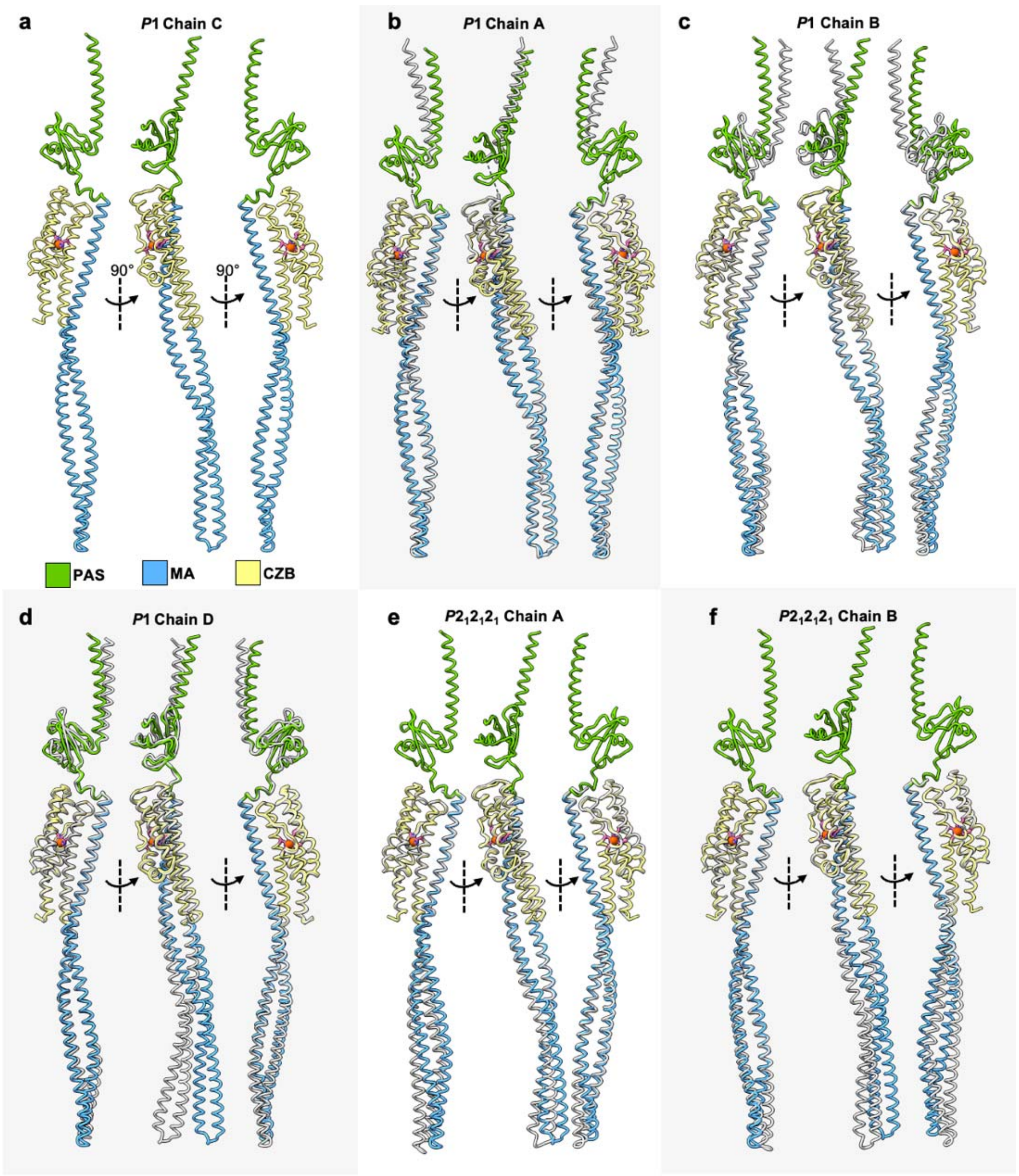

Supplemental Fig. 8. Overlays of TlpD chains a. Reference Chain C from the TlpD *P*1 crystal structure in three orientations, colored as in 1a, with the zinc ion shown in orange as an enlarged sphere for clarity. b-d. Chains A, B and D (white) from the TlpD *P*1 crystal structure are shown overlaid onto reference Chain C (colored by domain). e-f. Chains A and B from the $P2_12_12_1$ crystal structure are shown (white) overlaid onto reference Chain C (labeled by domain color) of

bioRxiv preprint doi: https://doi.org/10.64898/2026.01.16.699579; this version posted January 17, 2026. The copyright holder for this preprint (which was not certified by peer review) is the author/funder, who has granted bioRxiv a license to display the preprint in perpetuity. It is made available under aCC-BY 4.0 International license.

the TlpD *P*1 crystal structure. Residues 333-352 of the CZB domain were used in the alignments. See also Supplemental Table 2 for Cα RMSD values.

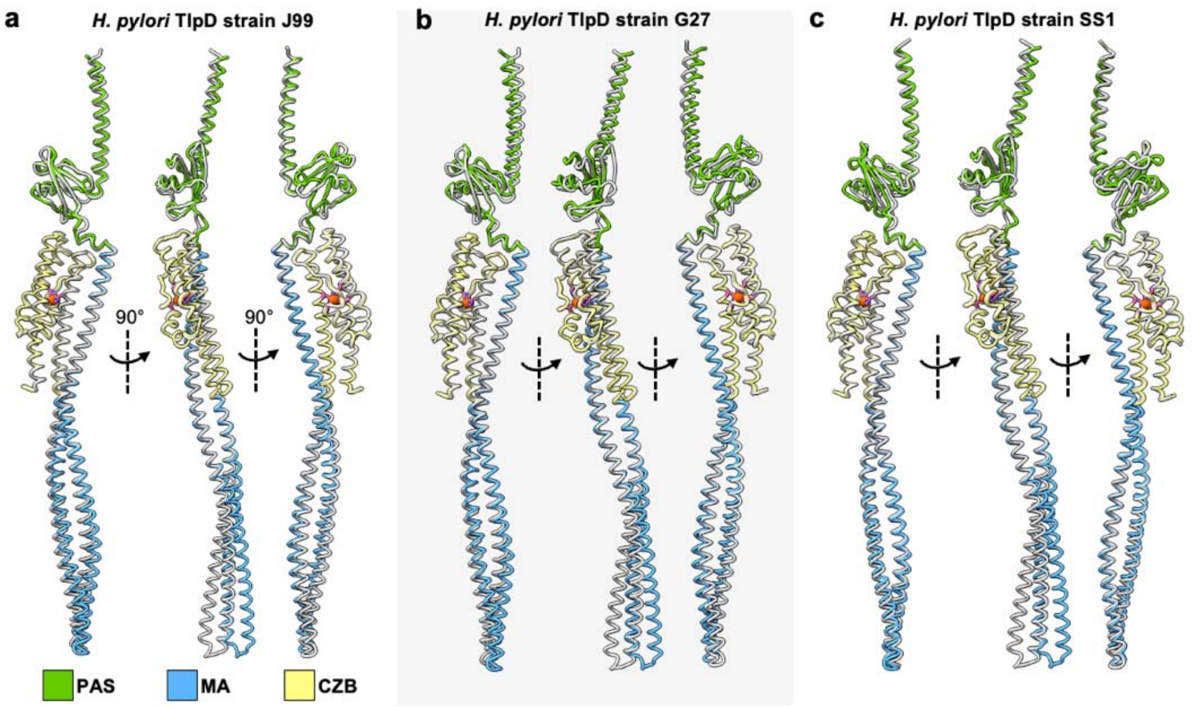

Supplemental Fig. 9. Comparisons with AlphaFold 3 models of TlpD from different *H. pylori* strains. Chain A (white) from AlphaFold 3 models of TlpD from *H. pylori* strain J99, G27 and SS1 respectively, were overlaid onto reference Chain C (domains colored as in 1a) from the TlpD *P*1 crystal structure based on alignment of residues 333–352 (CZB domain). AlphaFold 3 models were generated as homodimers with Chain A used for the overlays. See also Supplemental Table 2 for Cα RMSD values.

bioRxiv preprint doi: https://doi.org/10.64898/2026.01.16.699579; this version posted January 17, 2026. The copyright holder for this preprint (which was not certified by peer review) is the author/funder, who has granted bioRxiv a license to display the preprint in perpetuity. It is made available under aCC-BY 4.0 International license.

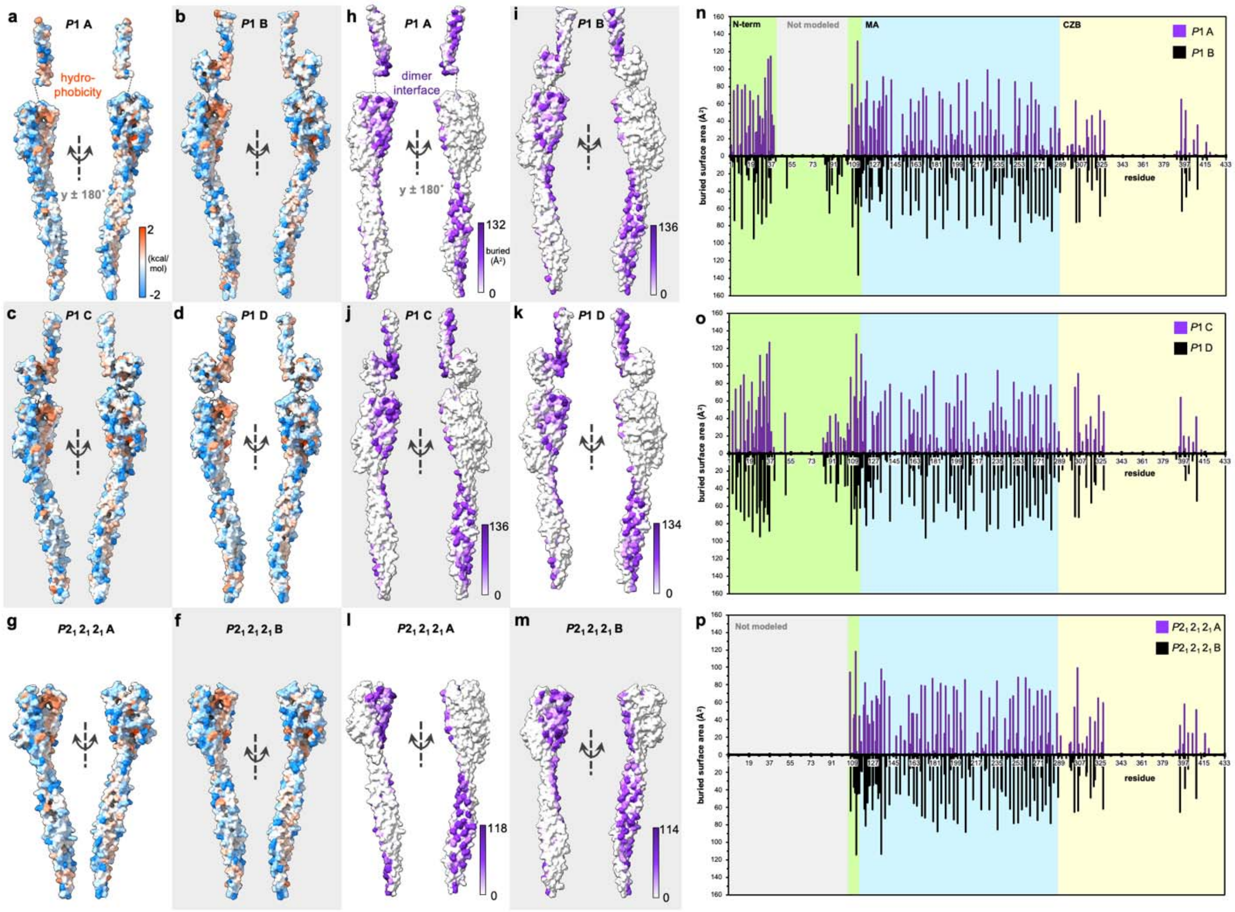

Supplemental Fig. 10. The homodimer interface of TlpD. a-d. *P*1 chains C and D colored by hydrophobicity using the Wimley-White hydrophobicity scale, which is based on the free energy associated with transitioning a peptide from an aqueous environment to a hydrophobic environment in units of kcal/mol. e-j. TlpD homodimer interface of all chains from the *P*1 (a-d) and $P2_1\,2_1\,2_1$ (e-f) crystal structures. k-m. Buried surface area per residue, for the *P*1 TlpD AB homodimer, CD homodimer, and $P2_1\,2_1\,2_1$ AB homodimer, as indicated. Plot regions are colored by domain as in 1a, or gray for regions not modeled.

bioRxiv preprint doi: https://doi.org/10.64898/2026.01.16.699579; this version posted January 17, 2026. The copyright holder for this preprint (which was not certified by peer review) is the author/funder, who has granted bioRxiv a license to display the preprint in perpetuity. It is made available under aCC-BY 4.0 International license.

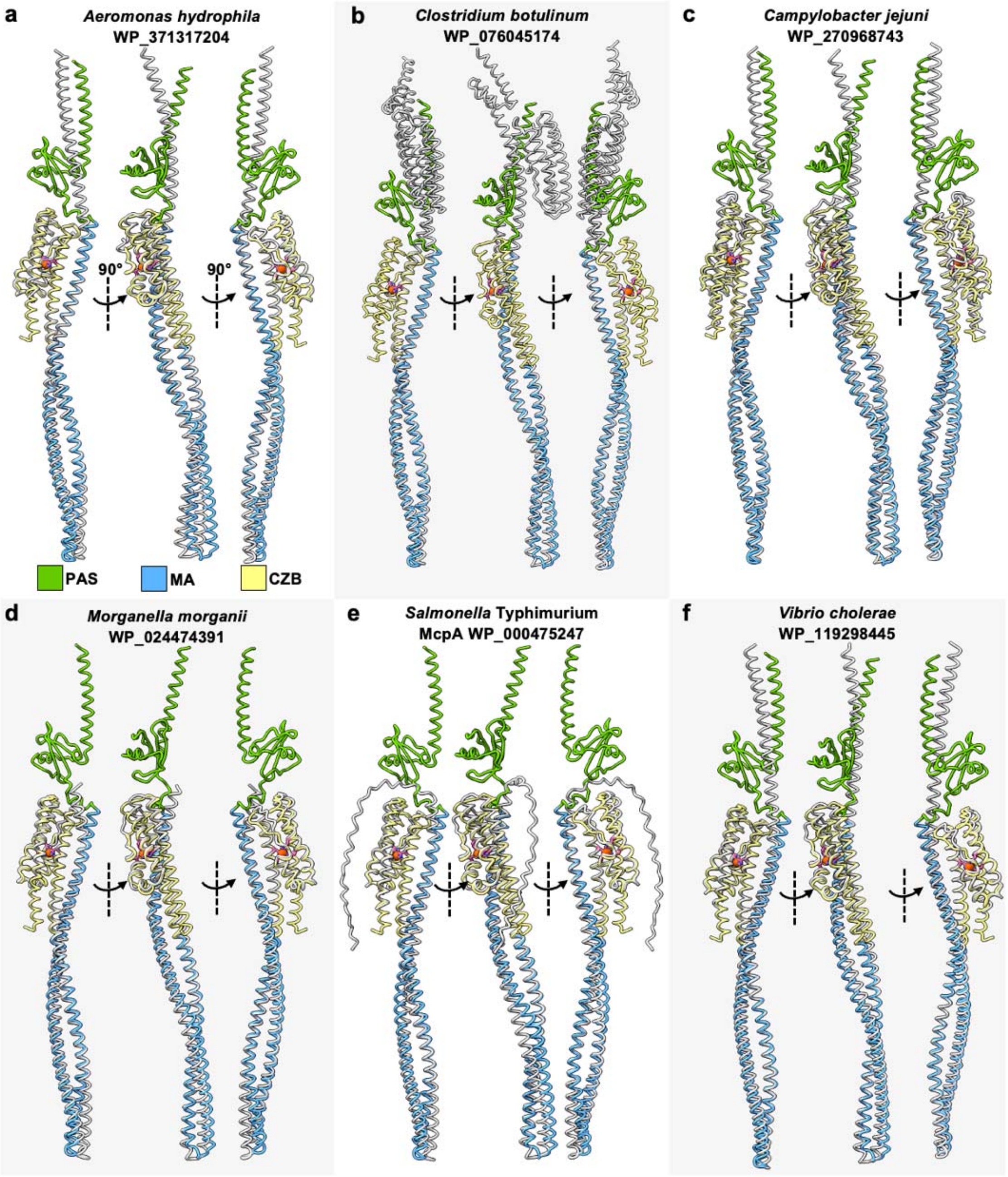

Supplemental Fig. 11. Comparisons with AlphaFold 3 models of TlpD orthologs. AlphaFold 3 models of six different TlpD homologs (white) from various bacterial families are shown overlaid onto reference Chain C (domains colored as in 1a) from the TlpD *P*1 crystal structure based on alignment of residues 333–352 (CZB domain). AlphaFold 3 models were generated as homodimers with Chain A used for the overlays. See also Supplemental Table 2 for Cα RMSD values.

bioRxiv preprint doi: https://doi.org/10.64898/2026.01.16.699579; this version posted January 17, 2026. The copyright holder for this preprint (which was not certified by peer review) is the author/funder, who has granted bioRxiv a license to display the preprint in perpetuity. It is made available under aCC-BY 4.0 International license.

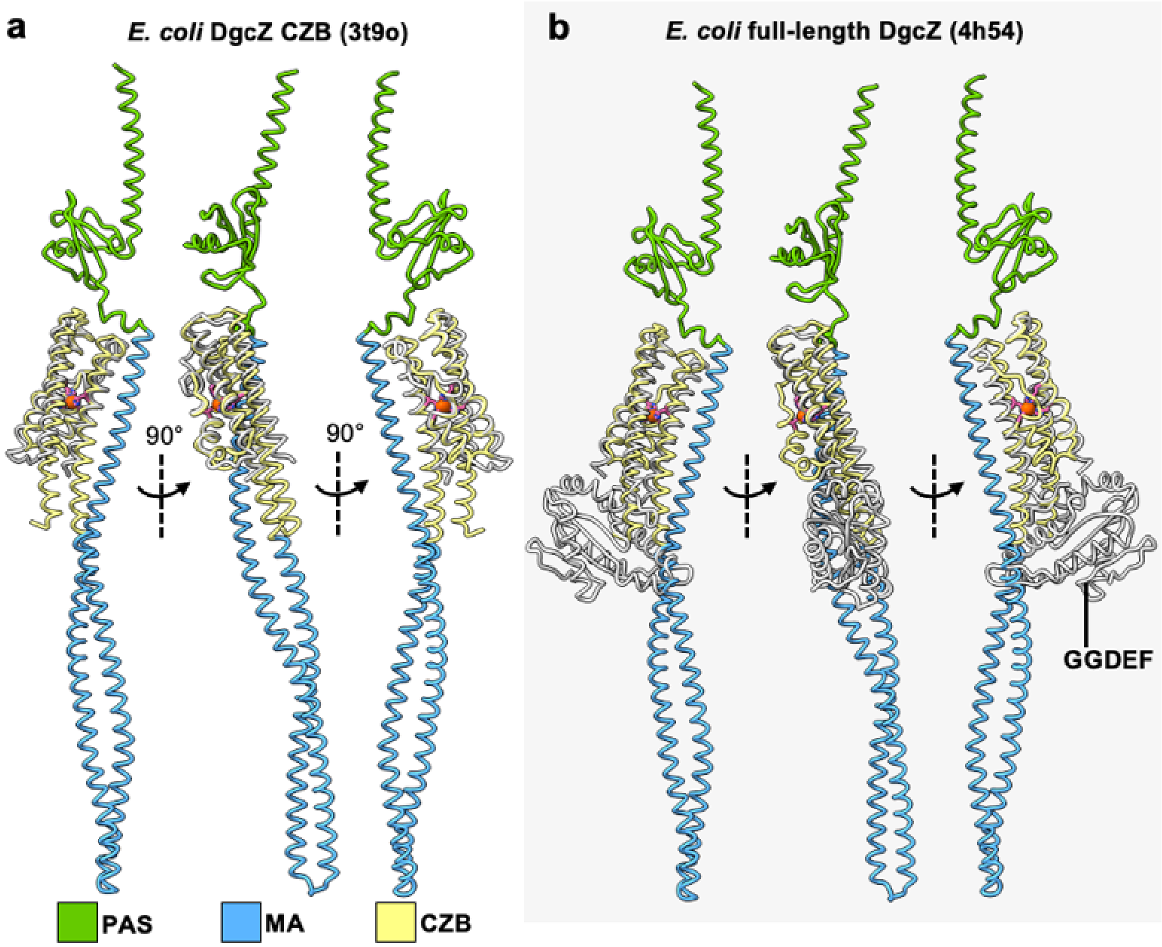

Supplemental Fig. 12. Comparisons of TlpD and DgcZ structures. a-c. Chain A (white) from the crystal structures of the *E. coli* DgcZ CZB fragment, and full-length structure of a C52A mutant, PDB ascension codes 3t9o and 4h54, respectively, are shown overlaid onto reference Chain C (domains colored as in 1a) from the TlpD *P*1 crystal structure based on alignment of residues 333-352 (CZB domain). See also Supplemental Table 2 for Cα RMSD values.

bioRxiv preprint doi: https://doi.org/10.64898/2026.01.16.699579; this version posted January 17, 2026. The copyright holder for this preprint (which was not certified by peer review) is the author/funder, who has granted bioRxiv a license to display the preprint in perpetuity. It is made available under aCC-BY 4.0 International license.

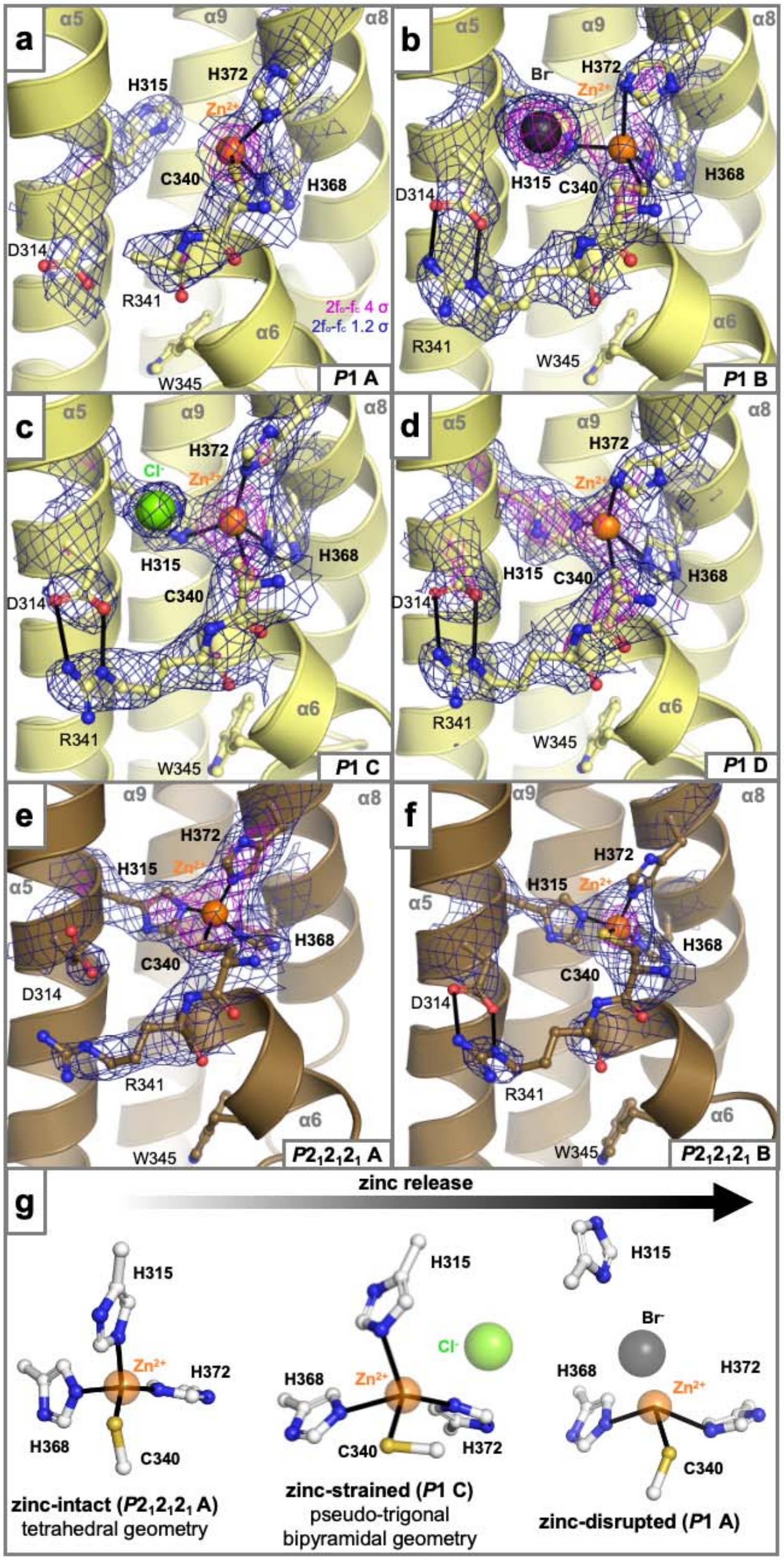

798

bioRxiv preprint doi: https://doi.org/10.64898/2026.01.16.699579; this version posted January 17, 2026. The copyright holder for this preprint (which was not certified by peer review) is the author/funder, who has granted bioRxiv a license to display the preprint in perpetuity. It is made available under aCC-BY 4.0 International license.

Supplemental Fig. 13. Zinc-binding site interactions in the TlpD crystal structures. a-f. The zinc-binding interactions are shown for respective chains, as indicated, with $2F_o$-F_c electron density at 1.2 (dark blue mesh) and 4 σ (pink mesh). Zinc ligation interactions are shown as thick black lines. For panel A, the Br^- atom present is omitted for clarity. g. Model showing how the zinc binding site geometry changes may relate to signaling state.

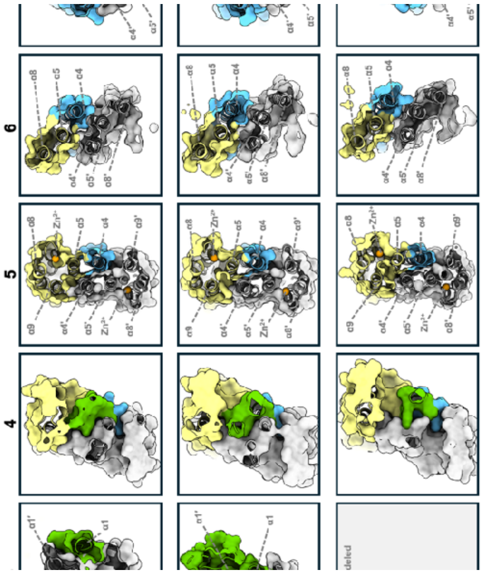

Supplemental Fig. 14. TlpD structures compared through the coiled-coil axis. a. Positions of cross-sectional slices taken at 24 Å intervals for each TlpD structure along the central coiled-coil axis are indicated with dashed lines. b. For each indicated position (1-10), top-down views are shown of the corresponding cross section in panel a. Each panel represents a 24 Å-thick slab, revealing changes in oligomeric architecture and interfaces along the length of the protein. Chains A and C of the *P*1 structure, and A of the $P2_12_12_1$ structure are colored by domain as in 1a, with partner chains in white.

bioRxiv preprint doi: https://doi.org/10.64898/2026.01.16.699579; this version posted January 17, 2026. The copyright holder for this preprint (which was not certified by peer review) is the author/funder, who has granted bioRxiv a license to display the preprint in perpetuity. It is made available under aCC-BY 4.0 International license.

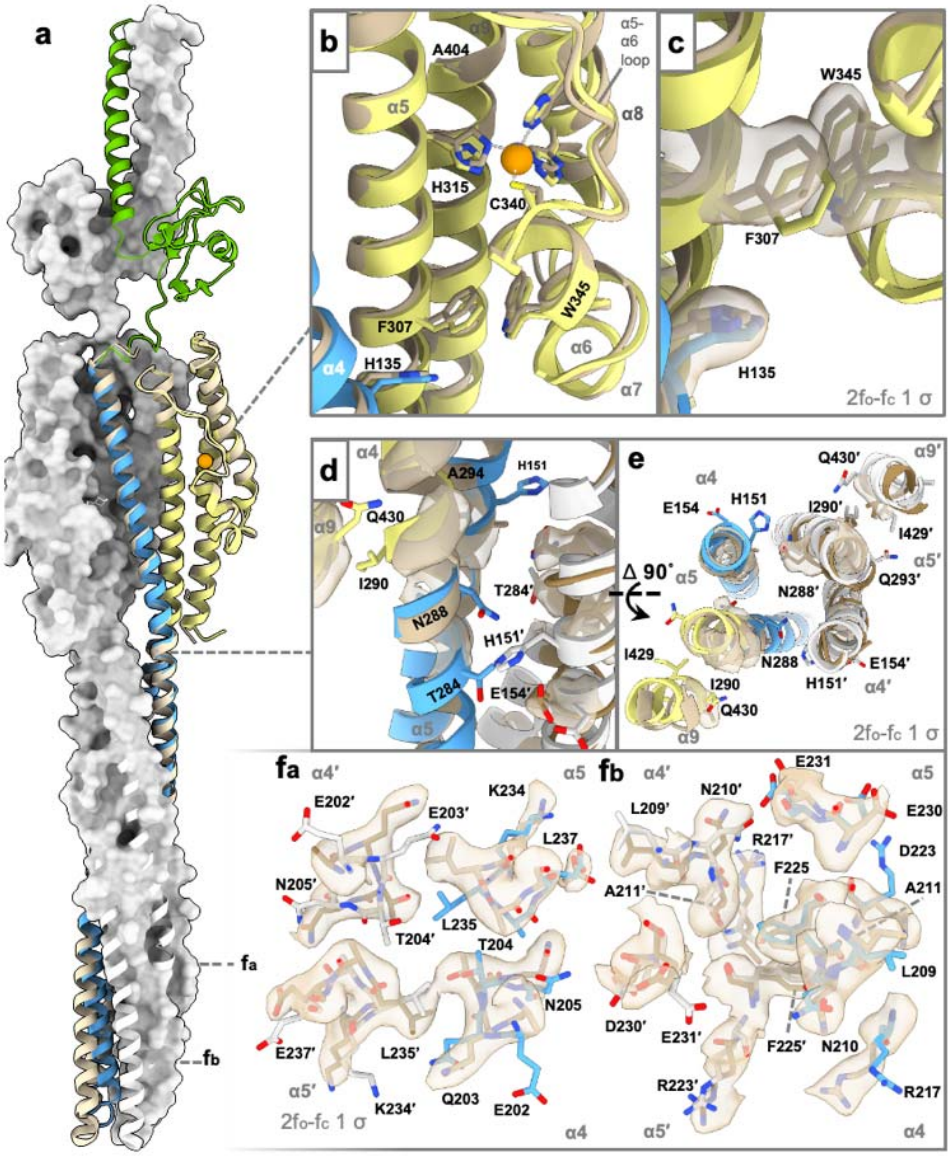

Supplemental Fig. 15. Comparison of zinc-intact and zinc-strained TlpD conformations. An overlay between the zinc-strained dimer (*P*1 C, colored by domain, as seen previously, *P*1 D in light gray) and zinc-intact dimer ($P2_12_12_1$ A in tan, B in brown) is shown. b-e. Close-up views of structural changes, as in Fig. 4. f. $2F_o$-F_c electron density is shown at 1 σ for selected residues of the zinc-intact conformation, which can be compared to Fig.4f.

bioRxiv preprint doi: https://doi.org/10.64898/2026.01.16.699579; this version posted January 17, 2026. The copyright holder for this preprint (which was not certified by peer review) is the author/funder, who has granted bioRxiv a license to display the preprint in perpetuity. It is made available under aCC-BY 4.0 International license.

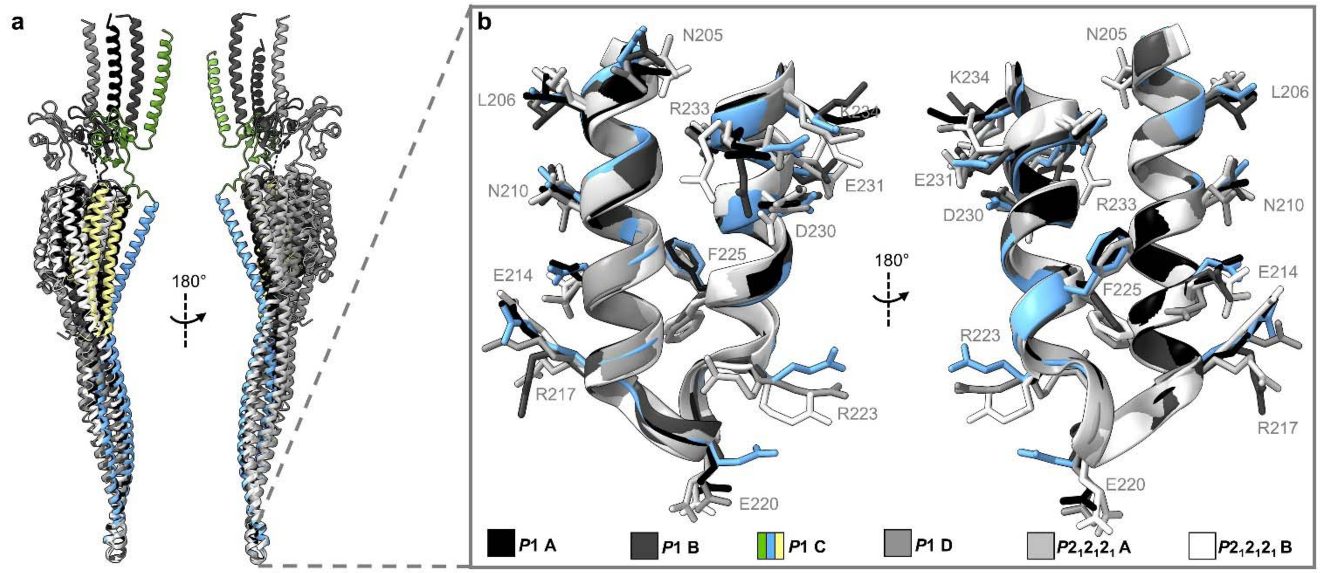

Supplemental Fig. 16. Overlays of the TlpD coiled-coil tips. a. Overall view of differences in the TlpD chains when overlaid based on only the tip resides E204-K234. Domains for *P*1 Chain C are colored as in 1a. b. Zoomed-in view of coiled-coil tip in two orientations, showing the local structurer of the tip is retained across the zinc-intact, zinc-strained, and zinc-disrupted conformations.

bioRxiv preprint doi: https://doi.org/10.64898/2026.01.16.699579; this version posted January 17, 2026. The copyright holder for this preprint (which was not certified by peer review) is the author/funder, who has granted bioRxiv a license to display the preprint in perpetuity. It is made available under aCC-BY 4.0 International license.

Supplemental Tables

Supplemental Table 1: Summary of crystallographic statistics.

Space Group	*P* 1	*P* 2_1 2_1 2_1
Cell dimensions and angle (a, b, c, α, β, γ) (Å, °)	61.0, 84.5, 90.7, 92.2, 90.4, 103.7	36.3, 127.8, 192.1, 90, 90, 90
Low resolution (Å) [a]	49.2	33.94
Resolution limit (each axis, Å)	2.454, 2.904, 3.276	3.065, 3.074, 2.922
Completeness (%) [a]	67.33 (10.47)	85.1 (19.7)
Total reflections	508149 (968)	155943 (8397)
Unique reflections	131548 (419)	27093 (1916)
Average I/σ [a]	2.29 (9.73)	4.18 (0.21)
R_{merge} [a]	0.66 (0.62)	0.1801 (5.515)
$CC_{1/2}$ [a]	0.85 (0.15)	0.997 (0.018)
R_{work} (%)	22.6	21.7
R_{free} (%)	29.2	30.1
Ramachandran favored, allowed, outliers (%)	95.3, 4.1, 0.6	82.3, 14.1, 3.6
Non-hydrogen atoms	12589	4979
Solvent atoms	37	9
Protein chains, residues	4, 1662	2, 648
Average B-factor of protein atoms ($Å^2$)	59.7	79.6
Average B-factor of solvent	27.0	30.6

bioRxiv preprint doi: https://doi.org/10.64898/2026.01.16.699579; this version posted January 17, 2026. The copyright holder for this preprint (which was not certified by peer review) is the author/funder, who has granted bioRxiv a license to display the preprint in perpetuity. It is made available under aCC-BY 4.0 International license.

atoms (Å^2)		
rms bond lengths (Å)	0.017	0.007
rms bond angles (°)	1.57	0.99
TLS groups	13	9
Molprobity clash score, percentile[77]	21.3, 75th	10.6, 97th

[a] Values in parentheses indicate statistics for the highest resolution shell.

Supplemental Table 2. Superposition of TlpD structures and models.

**Overlaid Structure/Model**	**Overlaid Chain**	**# Cα pairs**	**RMSD (Å)**
*P*1	A	368	2.673
*P*1[a]	A	20	0.766
*P*1	B	431	3.798
*P*1[a]	B	20	0.312
*P*1	D	430	3.426
*P*1[a]	D	20	0.362
$P2_12_12_1$	A	325	2.278
$P2_12_12_1$[a]	A	20	0.555
$P2_12_12_1$	B	323	2.099
$P2_12_12_1$[a]	B	20	0.551
J99 TlpD WP_000467796[b,c]	A	431	3.033
J99 TlpD WP_000467796[a,b,c]	A	20	0.412

bioRxiv preprint doi: https://doi.org/10.64898/2026.01.16.699579; this version posted January 17, 2026. The copyright holder for this preprint (which was not certified by peer review) is the author/funder, who has granted bioRxiv a license to display the preprint in perpetuity. It is made available under aCC-BY 4.0 International license.

J99 TlpD WP_000467796 [b,c]	B	431	2.223
J99 TlpD WP_000467796 [a,b,c]	B	20	0.365
G27 TlpD WP_434671832 [b,c]	A	431	2.525
G27 TlpD WP_434671832 [a,b,c]	A	20	0.401
G27 TlpD WP_434671832 [b,c]	B	431	2.271
G27 TlpD WP_434671832 [a,b,c]	B	20	0.400
SS1 TlpD WP_077232132 [b,c]	A	431	3.033
SS1 TlpD WP_077232132 [a,b,c]	A	20	0.412
SS1 TlpD WP_077232132 [b,c]	B	431	2.589
SS1 TlpD WP_077232132 [a,b,c]	B	20	0.410
Aeromonas hydrophila WP_371317204 [b,c]	A	344	6.556
Aeromonas hydrophila WP_371317204 [a.b,c]	A	18	0.610
Aeromonas hydrophila WP_371317204 [b,c]	B	344	6.741
Aeromonas hydrophila WP_371317204 [a.b,c]	B	18	0.606
Campylobacter jejuni WP_270968743 [b,c]	A	357	7.703
Campylobacter jejuni WP_270968743 [a.b,c]	A	16	0.663
Campylobacter jejuni WP_270968743 [b,c]	B	357	7.577
Campylobacter jejuni WP_270968743 [a.b,c]	B	16	0.667
Clostridium botulinum WP_076045174 [b,c]	A	375	30.768
Clostridium botulinum WP_076045174 [a.b,c]	A	13	0.640
Clostridium botulinum WP_076045174 [b,c]	B	375	30.932
Clostridium botulinum WP_076045174 [a.b,c]	B	13	0.647
Morganella morganii WP_024474391 [b,c]	A	342	17.192

bioRxiv preprint doi: https://doi.org/10.64898/2026.01.16.699579; this version posted January 17, 2026. The copyright holder for this preprint (which was not certified by peer review) is the author/funder, who has granted bioRxiv a license to display the preprint in perpetuity. It is made available under aCC-BY 4.0 International license.

Morganella morganii WP_024474391 [a.b,c]	A	18	0.814
Morganella morganii WP_024474391 [b,c]	B	342	17.782
Morganella morganii WP_024474391 [a.b,c]	B	18	0.812
Salmonella Typhimurium McpA WP_000475247 [b,c]	A	347	17.521
Salmonella Typhimurium McpA WP_000475247 [a.b,c]	A	18	0.964
Salmonella Typhimurium McpA WP_000475247 [b,c]	B	347	17.181
Salmonella Typhimurium McpA WP_000475247 [a.b,c]	B	18	0.966
Vibrio cholerae WP_119298445 [b,c]	A	359	4.919
Vibrio cholerae WP_119298445 [a.b,c]	A	19	0.851
Vibrio cholerae WP_119298445 [b,c]	B	359	5.002
Vibrio cholerae WP_119298445 [a.b,c]	B	19	0.855
E. coli DgcZ 4h54	A	119	6.613
E. coli DgcZ 4h54 [a]	A	6	1.355
E. coli DgcZ 4h54	B	116	17.182
E. coli DgcZ 4h54 [a]	B	6	1.363
E. coli DgcZ 3t9o	A	105	8.885
E. coli DgcZ 3t9o [a]	A	8	0.627
E. coli DgcZ 3t9o	B	112	4.632
E. coli DgcZ 3t9o [a]	B	12	0.502
P1	A (tip)	31	0.528
P1	B (tip)	31	0.423

bioRxiv preprint doi: https://doi.org/10.64898/2026.01.16.699579; this version posted January 17, 2026. The copyright holder for this preprint (which was not certified by peer review) is the author/funder, who has granted bioRxiv a license to display the preprint in perpetuity. It is made available under aCC-BY 4.0 International license.

$P1$	D (tip)	31	0.389
$P2_12_12_1$	A (tip)	31	0.670
$P2_12_12_1$	B (tip)	31	0.737

[*] All overlays use *P*1 C as the reference chain.

[a] When choosing a select number of reference residues for overlays, these residues were 333-352 with an iteration cutoff distance of 2.0.

[b] The AlphaFold3 server was used to generate homodimers of these constructs based on sequence.

[c] This overlay used the stated NCBI protein sequence identifier to determine the sequence input for AlphaFold 3 modeling.

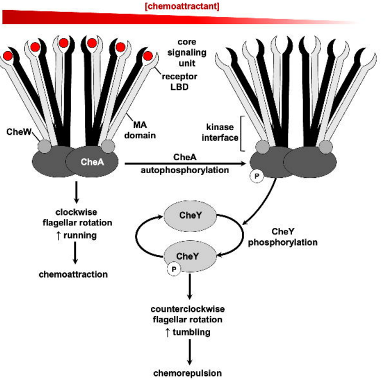
[chemoattractant]
core
signaling
unit
receptor
LBD
MA
domain
CheW
CheA
kinase
interface
CheA
autophosphorylation
P
clockwise
flagellar rotation
↑ running
chemoattraction
CheY
CheY
P
CheY
phosphorylation
counterclockwise
flagellar rotation
↑ tumbling
chemorepulsion

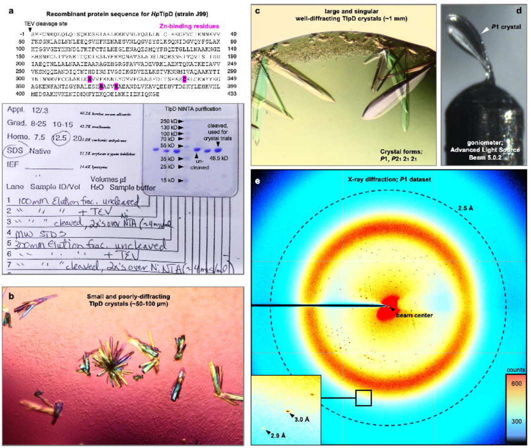
a
Recombinant protein sequence for HpTlpD (strain J99)
TEV cleavage site
Zn-binding residues
TlpD NiNTA purification
250 kD
130 kD
100 kD
70 kD
55 kD
35 kD
25 kD
15 kD
cleaved, used for crystal trials
un-cleaved
48.5 kD
b
Small and poorly-diffracting TlpD crystals (~50-100 µm)
c
large and singular well-diffracting TlpD crystals (~1 mm)
Crystal forms: P1, P2₁ 2₁ 2₁
d
P1 crystal
goniometer; Advanced Light Source Beam 5.0.2
e
X-ray diffraction; P1 dataset
2.5 Å
beam center
3.0 Å
2.9 Å
counts
600
300

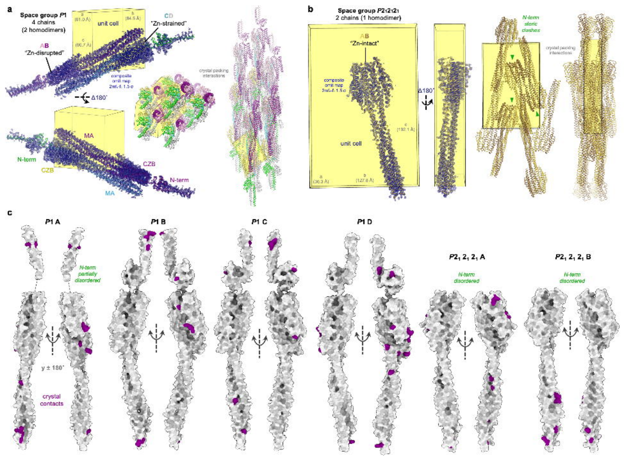
a
Space group P1
4 chains
(2 homodimers)
a (61.0 Å)
b (84.5 Å)
c (90.7 Å)
unit cell
CD
"Zn-strained"
AB
"Zn-disrupted"
composite omit map 2mFo-Fc 1.5 σ
Δ180°
crystal packing interactions
MA
N-term
CZB
CZB
N-term
MA
b
Space group P2₁2₁2₁
2 chains (1 homodimer)
AB
"Zn-intact"
composite omit map 2mFo-Fc 1.5 σ
Δ180°
c (192.1 Å)
unit cell
a (36.3 Å)
b (127.8 Å)
N-term steric clashes
crystal packing interactions
c
P1 A
P1 B
P1 C
P1 D
P2₁ 2₁ 2₁ A
P2₁ 2₁ 2₁ B
N-term partially disordered
N-term disordered
N-term disordered
y ± 180°
crystal contacts

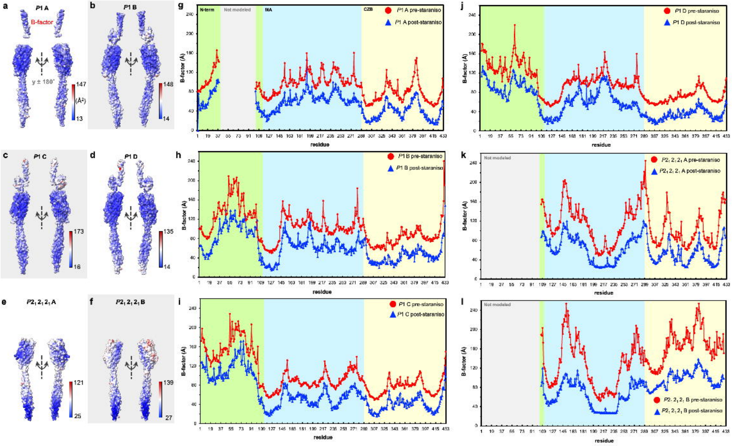

a
P1 A
B-factor
y ± 180°
147
(Å²)
13
b
P1 B
148
14
c
P1 C
173
16
d
P1 D
135
14
e
P2₁ 2₁ 2₁ A
121
25
f
P2₁ 2₁ 2₁ B
139
27
g
N-term
Not modeled
MA
CZB
P1 A pre-staraniso
P1 A post-staraniso
B-factor (A)
residue
h
P1 B pre-staraniso
P1 B post-staraniso
i
P1 C pre-staraniso
P1 C post-staraniso
j
P1 D pre-staraniso
P1 D post-staraniso
k
Not modeled
P2₁ 2₁ 2₁ A pre-staraniso
P2₁ 2₁ 2₁ A post-staraniso
l
Not modeled
P2₁ 2₁ 2₁ B pre-staraniso
P2₁ 2₁ 2₁ B post-staraniso

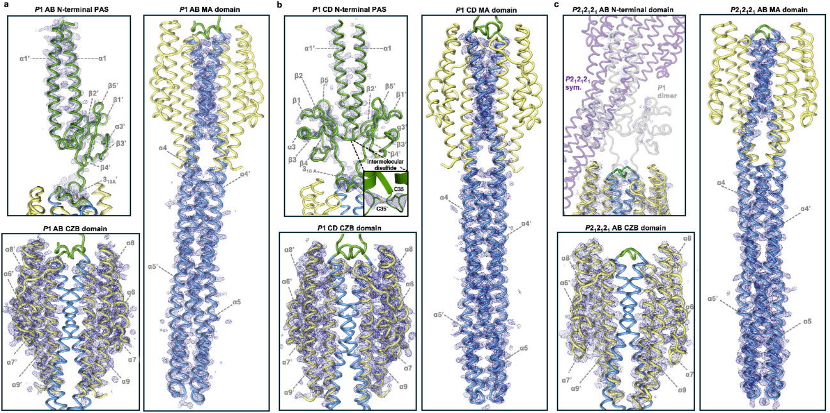
a
P1 AB N-terminal PAS
P1 AB MA domain
P1 AB CZB domain
b
P1 CD N-terminal PAS
intermolecular disulfide
C35
C35'
P1 CD MA domain
P1 CD CZB domain
c
P2₁2₁2₁ AB N-terminal domain
P2₁2₁2₁ sym.
P1 dimer
P2₁2₁2₁ AB MA domain
P2₁2₁2₁ AB CZB domain
α1'
α1
β2'
β5'
β1'
α3'
β3'
β4'
α4
α4'
α5'
α5
α8'
α8
α6'
α6
α7'
α7
α9'
α9

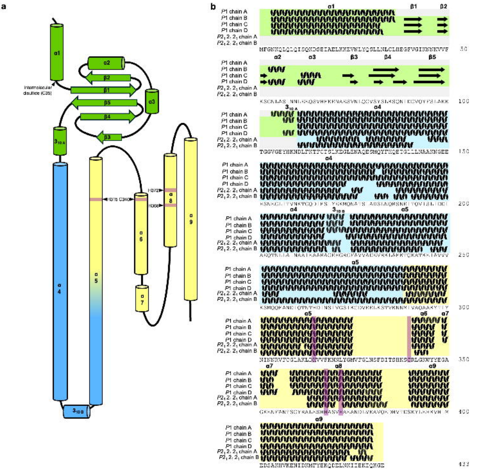
a
b
α1
Intermolecular disulfide (C35)
α2
β2
β1
β5
β4
β3
α3
3 10 A
α4
α5
α6
α7
α8
α9
3 10 B
H316 C340
H372
H368
P1 chain A
P1 chain B
P1 chain C
P1 chain D
P2 1 2 1 2 1 chain A
P2 1 2 1 2 1 chain B
50
100
150
200
250
300
350
400
433

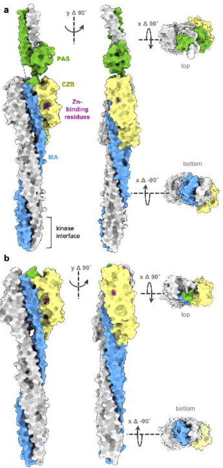

a
y Δ 90˚
PAS
CZB
Zn-binding residues
MA
kinase interface
x Δ 90˚
top
bottom
x Δ -90˚
b
y Δ 90˚
x Δ 90˚
top
bottom
x Δ -90˚

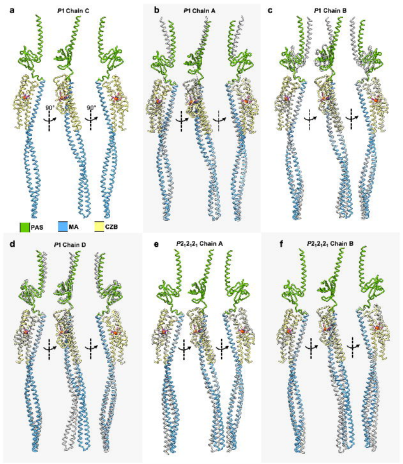

a
$P1$ Chain C
90°
90°
PAS
MA
CZB
b
$P1$ Chain A
c
$P1$ Chain B
d
$P1$ Chain D
e
$P2_12_12_1$ Chain A
f
$P2_12_12_1$ Chain B

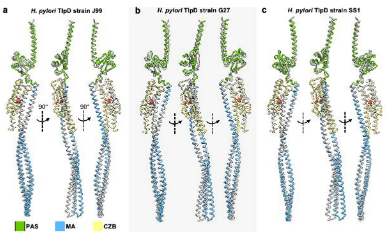
a
H. pylori TlpD strain J99
90°
90°
b
H. pylori TlpD strain G27
c
H. pylori TlpD strain SS1
PAS
MA
CZB

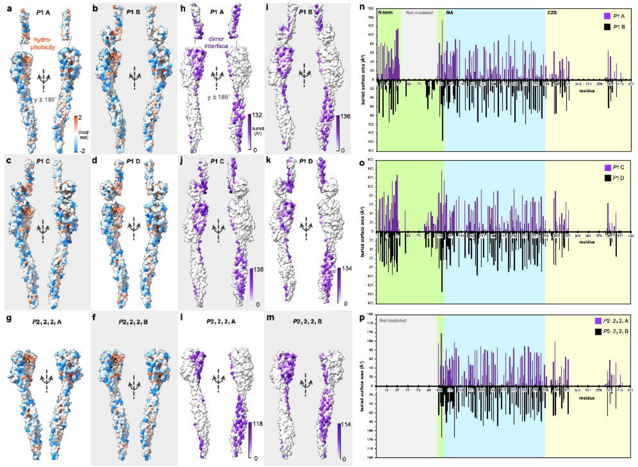

a
P1 A
hydro-phobicity
y ± 180˚
2
(kcal/mol)
-2
b
P1 B
c
P1 C
d
P1 D
g
P2₁2₁2₁ A
f
P2₁2₁2₁ B
h
P1 A
dimer interface
y ± 180˚
132
buried (Å²)
0
i
P1 B
136
0
j
P1 C
136
0
k
P1 D
134
0
l
P2₁2₁2₁ A
118
0
m
P2₁2₁2₁ B
114
0
n
N-term
Not modeled
MA
CZB
P1 A
P1 B
buried surface area (Å²)
residue
o
P1 C
P1 D
buried surface area (Å²)
residue
p
Not modeled
P2₁2₁2₁ A
P2₁2₁2₁ B
buried surface area (Å²)
residue

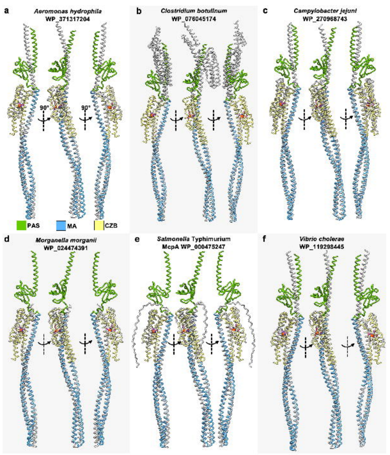

a
Aeromonas hydrophila
WP_371317204
90°
90°
b
Clostridium botulinum
WP_076045174
c
Campylobacter jejuni
WP_270968743
PAS
MA
CZB
d
Morganella morganii
WP_024474391
e
Salmonella Typhimurium
McpA WP_000475247
f
Vibrio cholerae
WP_119298445

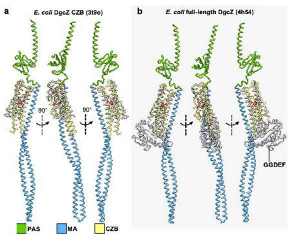

a
E. coli DgcZ CZB (3t9o)
90°
90°
b
E. coli full-length DgcZ (4h54)
GGDEF
PAS
MA
CZB

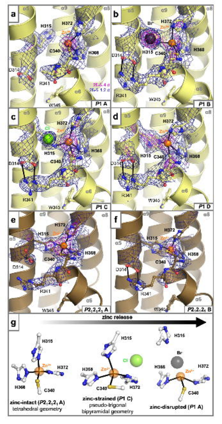

a
H315
H372
Zn2+
C340
H368
D314
R341
W345
P1 A
b
Br
H372
Zn2+
H315
C340
H368
D314
R341
W345
P1 B
c
Cl
Zn2+
H372
H315
C340
H368
D314
R341
W345
P1 C
d
Zn2+
H372
H315
C340
H368
D314
R341
W345
P1 D
e
H372
H315
C340
H368
D314
R341
W345
P2₁2₁2₁ A
f
H372
H315
C340
H368
D314
R341
W345
P2₁2₁2₁ B
g
zinc release
H315
Zn2+
H372
H368
C340
zinc-intact (P2₁2₁2₁ A)
tetrahedral geometry
H315
Cl
H368
Zn2+
C340
H372
zinc-strained (P1 C)
pseudo-trigonal
bipyramidal geometry
H315
Br
H368
H372
Zn2+
C340
zinc-disrupted (P1 A)

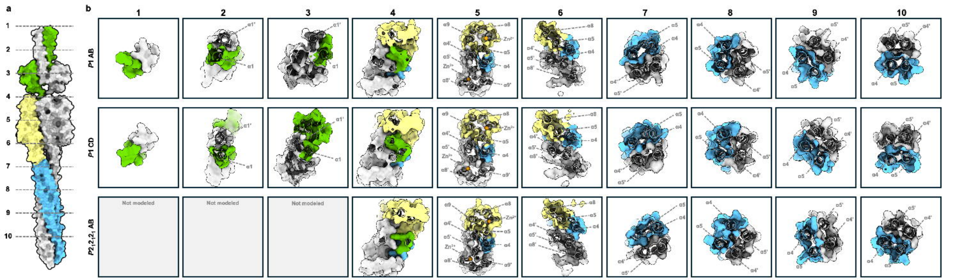

a
b
1
2
3
4
5
6
7
8
9
10
P1 AB
P1 CD
P2₁2₁2₁ AB
Not modeled
Not modeled
Not modeled
α1'
α1
α9
α8
Zn²⁺
α4'
α5'
α8'
α9'
α4
α5

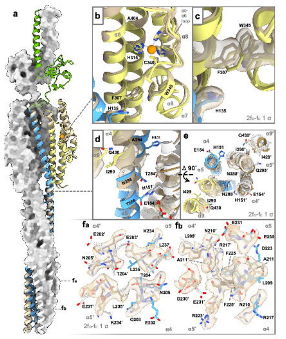

a
b
c
d
e
fa
fb
A404
α5
α8
H315
C340
W345
F307
H135
α4
α6
α7
2fo-fc 1 σ
A294
Q430
I290
N288
T284
T284'
H151'
E154'
Δ 90°
E154
H151
Q430'
I290'
N288'
I429'
Q293'
α5'
α9'
α4'
I429
α9
E202'
E203'
K234
L237
N205'
L235
T204'
T204
N205
E237'
L235'
K234'
Q203
E202
α5'
α4
E231
L209'
N210'
R217'
E230
D223
A211'
F225
A211
L209
D230'
E231'
F225'
N210
R223'
R217

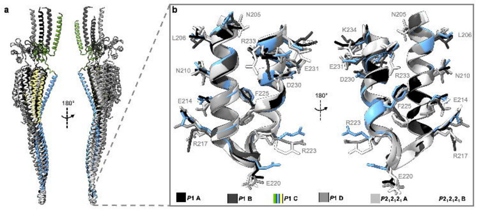
a
180°
b
N205
L206
R233
N210
E231
D230
F225
E214
R217
R223
E220
K234
P1 A
P1 B
P1 C
P1 D
P2₁2₁2₁ A
P2₁2₁2₁ B
